# Comparison of GENCODE and RefSeq gene annotation and the impact of reference geneset on variant effect prediction

**DOI:** 10.1186/1471-2164-16-S8-S2

**Published:** 2015-06-18

**Authors:** Adam Frankish, Barbara Uszczynska, Graham RS Ritchie, Jose M Gonzalez, Dmitri Pervouchine, Robert Petryszak, Jonathan M Mudge, Nuno Fonseca, Alvis Brazma, Roderic Guigo, Jennifer Harrow

**Affiliations:** 1 Wellcome Trust Sanger Institute, Wellcome Trust Genome Campus, Hinxton, Cambridge, CB10 1SA, UK; 2 Centre for Genomic Regulation, Barcelona, Catalonia, Spain; 3 European Molecular Biology Laboratory, European Bioinformatics Institute, Wellcome Trust Genome Campus, Hinxton, Cambridge, CB10 1SD, UK; 4 Faculty of Bioengineering and Bioinformatics, 119992 Moscow GSP-2, Leninskie Gory, Moscow State University 1-73, Russia

## Abstract

**Background:**

A vast amount of DNA variation is being identified by increasingly large-scale exome and genome sequencing projects. To be useful, variants require accurate functional annotation and a wide range of tools are available to this end. McCarthy et al recently demonstrated the large differences in prediction of loss-of-function (LoF) variation when RefSeq and Ensembl transcripts are used for annotation, highlighting the importance of the reference transcripts on which variant functional annotation is based.

**Results:**

We describe a detailed analysis of the similarities and differences between the gene and transcript annotation in the GENCODE and RefSeq genesets. We demonstrate that the GENCODE Comprehensive set is richer in alternative splicing, novel CDSs, novel exons and has higher genomic coverage than RefSeq, while the GENCODE Basic set is very similar to RefSeq. Using RNAseq data we show that exons and introns unique to one geneset are expressed at a similar level to those common to both. We present evidence that the differences in gene annotation lead to large differences in variant annotation where GENCODE and RefSeq are used as reference transcripts, although this is predominantly confined to non-coding transcripts and UTR sequence, with at most ~30% of LoF variants annotated discordantly. We also describe an investigation of dominant transcript expression, showing that it both supports the utility of the GENCODE Basic set in providing a smaller set of more highly expressed transcripts and provides a useful, biologically-relevant filter for further reducing the complexity of the transcriptome.

**Conclusions:**

The reference transcripts selected for variant functional annotation do have a large effect on the outcome. The GENCODE Comprehensive transcripts contain more exons, have greater genomic coverage and capture many more variants than RefSeq in both genome and exome datasets, while the GENCODE Basic set shows a higher degree of concordance with RefSeq and has fewer unique features. We propose that the GENCODE Comprehensive set has great utility for the discovery of new variants with functional potential, while the GENCODE Basic set is more suitable for applications demanding less complex interpretation of functional variants.

## Background

Falling costs have led to a surge in the number of complete human exomes and genome sequences available. Large scale sequencing projects such as the 1000 Genomes Project [1], UK10K [2,3] and NHLBI Go Exome Sequencing Project (ESP) [4] are being followed by even larger projects such as the 100,000 Genomes Project [5]. While such datasets are of great interest to both researchers and clinicians, their ultimate value depends not on the number of variants identified, but rather on their functional interpretation or 'annotation'. An obvious starting point in the annotation process is to judge whether the variant lies in a genic or intergenic region and, if it is the former, whether it is found in coding (CDS) or non-coding sequence. In fact, any information placed onto the genome sequence can theoretically be used to annotate variation. For example, while variant annotation pipelines such as Ensembl Variant Effect Predictor (VEP) [6], Annovar [7], VAAST [8] and VAT [9] distinguish between CDS and untranslated regions (UTRs) of transcripts, they also consider whether variants fall within regions critical to the splicing process. However, as well as describing the location of variants, pipelines must also try and interpret their biological consequences. For CDS variants, stop codon gain or loss events and frameshifting due to indels may be identified and tools such as SIFT [10] and PolyPhen-2 [11] can infer the nature of any amino acid changes due to missense substitutions and give an estimation of their deleteriousness.

Clearly, the transcripts used for variant annotation are critically important to the process. Recently, Macarthy et al. [12] reported a significant divergence in the annotation of the same set of variants when two different transcript sets ('genesets'), GENCODE [13,14] and RefSeq [15], were used. While they share many similarities, the disparity in variant annotation observed is nonetheless driven by fundamental differences between these genesets. The GENCODE consortium was established to produce a reference gene annotation for the ENCODE project [16,17]. This geneset aims to capture the full extent of transcriptional complexity, including long non-coding RNAs (lncRNAs), pseudogenes and small RNAs alongside protein-coding genes, and all transcripts that are associated with these loci. GENCODE combines manual annotation by the HAVANA group [18] with computational annotation by Ensembl [19], although 93.4% of transcripts associated with protein-coding genes are either solely manually annotated or identical in both manual and automated annotation in release v21. The extensive use of manual curation in GENCODE affords the use of a wider range of functionally descriptive gene and transcript 'biotypes'. Pertinently, GENCODE can annotate transcripts containing a premature stop codon as 'nonsense mediated decay' (NMD) models on the basis that they are likely to undergo degradation by RNA surveillance pathways [20]. GENCODE is also subjected to ongoing computational validation by other groups within the consortium (using tools such as Pseudopipe [21], Retrofinder [22], PhyloCSF [23], APPRIS [24]) while putative models can also be targeted for experimental confirmation [25]. The GENCODE geneset is publically available via http://www.gencodegenes.org, and it can be visualised using the VEGA [18], Ensembl [19] and UCSC [26] portals. GENCODE is the default annotation used by the Ensembl project, and the terms 'Ensembl annotation' and 'GENCODE annotation' are thus synonymous when referring to human.

The widely used RefSeq geneset is produced by NCBI [15]. It can also be visualised using the UCSC and Ensembl browsers, and downloaded from http://www.ncbi.nlm.nih.gov/RefSeq. The RefSeq human protein-coding transcript set also contains a significant manually annotated component. However, it also incorporates a large number of computationally-predicted transcripts; in NCBI Homo sapiens Annotation Release 106 ~31% of transcripts within protein-coding genes are now categorised as REVIEWED, ~20% as VALIDATED and 2% as PROVISIONAL, with <1% as PREDICTED, INFERRED and ~45% as MODEL. Additional file 1: Figure S1 shows the RefSeq annotation of the human BRCA1 locus, which includes predicted protein-coding 'XM' models alongside manually curated protein-coding 'NM' transcripts and non-coding 'NR' transcripts.

Historically, the GENCODE geneset has been richer in alternative splicing (AS) than RefSeq [14]. It also differs in the way it represents transcripts based on truncated evidence, i.e. where the RNA obtained from sequencing is inferred to be a portion of the actual RNA molecule. Whereas RefSeq extend all transcripts at a locus sharing the same first and final exon to use the same transcription start and end site, GENCODE only extend a transcript as far as the supporting evidence allows. As such, GENCODE does not predict gene structures for which there is no or incomplete supporting evidence, and this geneset contains many truncated transcripts (see Additional file 2: Figure S2); all such transcripts are clearly marked as such in genome browsers and GTF file with a start/end not found tag.

Here, we present a detailed comparison of the most recent versions of GENCODE (v21) and RefSeq (Release 67) in order to identify the similarities and differences between the transcripts, exons and the CDSs they encode. We analyse the expression profiles of transcripts unique to both the GENCODE and RefSeq genesets as well as those common to both, and discuss how this affects the utility of both sets in variant annotation. We then compare the effect of using different genesets in the annotation of two large variant sets mapped to the latest version of the human reference genome (GRCh38). Finally, we describe an investigation of the use of RNAseq data to provide a biological basis for reducing complexity of the GENCODE transcript set. We did not include the alternative geneset Aceview [27] in this analysis, as its human gene model annotation does not appear to have been updated since 2007, well before the release of GRCh38. Furthermore, previous analysis identified several confounding features, such as confusing locus definitions and the addition of a CDS to almost all transcripts [14].

## Results

### Comparison of GENCODE and RefSeq annotated transcripts

To quantify the differences between the GENCODE and RefSeq genesets, we investigated the general properties of transcripts from protein-coding genes that map to the reference human genome (GRCh38). Alternative splicing is the major source of transcriptional diversity within protein-coding genes, and this can occur in three ways: (1) through the 'skipping' of exons, (2) through the incorporation of additional exons, and (3) via the use of alternative splice sites within the same exon. Further diversity is also provided by the existence of transcripts that have 'retained', i.e. haven't spliced out, particular introns [28]. We have used four genesets for this analysis: GENCODE Comprehensive, GENCODE Basic, and two sets we define as RefSeq NXR and RefSeq NR. The former contains all manually curated NM and NR transcripts, and all XM and XR transcripts at protein-coding genes while the latter contains only manually curated transcripts. GENCODE Basic is a subset of GENCODE Comprehensive, containing only full-length protein-coding transcripts, while RefSeq NR is a subset of RefSeq NXR; further details are provided in Table 1.

**Table 1:** Definition of Geneset provenance

Geneset	Provenance
GENCODE Comprehensive	All transcripts at protein-coding genes. Includes transcripts with NMD, retained_intron and processed_transcript biotypes.

GENCODE Basic	Only full-length, protein-coding transcripts at protein-coding genes.

RefSeq NXR	All RefSeq transcripts at protein-coding genes. Includes manually annotated NM, NR and automated XM transcripts.

RefSeq NR	Only manually-annotated transcripts at protein-coding genes. Includes NM and NR transcripts

Transcript functional biotypes and source e.g. manual or automated annotation, for the four genesets used in this study.

**Figure 1:**
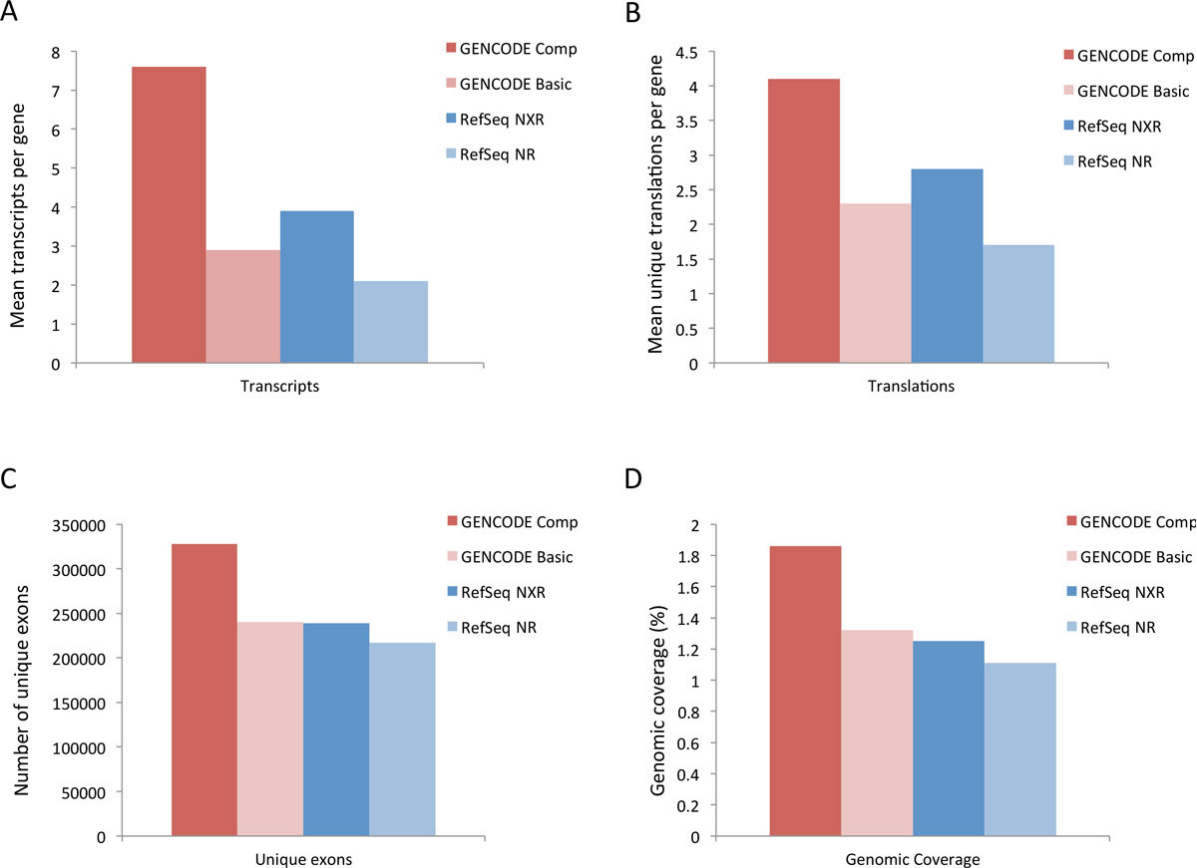
**General properties of GENCODE and RefSeq protein-coding genes**. A) Mean number of alternatively spliced transcripts per multi-exon protein-coding locus B) Mean number of unique CDS per multi-exon protein-coding locus C) Mean number of unique (non-redundant) exons per multi-exon protein-coding locus D) Percentage genomic coverage of unique (non-redundant) exons at multi-exon protein-coding loci

Figure 1A shows that the GENCODE Comprehensive geneset has almost twice as many AS transcripts per multi-exon protein-coding locus as RefSeq NXR; while the GENCODE Basic set has more than RefSeq NR. GENCODE Comprehensive also has the highest number of unique translations per locus followed by RefSeq NXR, GENCODE Basic and RefSeq NR (Figure 1B). The difference between the total numbers of transcripts and unique translations, in the GENCODE Comprehensive set is, in part, due to the presence of transcripts without translations, in particular those classed as 'retained introns' or 'processed transcripts' (which are typically based on truncated RNA evidence, such that a CDS cannot be annotated with confidence). However, it also reflects the existence of alternative splicing events limited to the 5' and more rarely 3' UTRs, i.e. where translation is unaffected. By definition, the GENCODE Basic set does not contain transcripts without translations, and as such all differences between the numbers of total transcripts and unique translations reflect AS with the 5' and 3' UTR. GENCODE Comprehensive also has the highest number of non-redundant exons, and again RefSeq NR has the fewest (Figure 1C). This is reflected in the total genomic coverage of unique exons shown in Figure 1D. Altogether this analysis suggests that the GENCODE Comprehensive set is larger and represents more transcriptional complexity than RefSeq. It is also notable that the RefSeq NXR set contains more AS transcripts and unique translations than the GENCODE Basic set despite containing fewer unique exons. It can thus be assumed that the additional transcripts and translations in the former result from the capture of novel exon-skipping events or combinations of exons rather than from the presence of novel exons.

**Figure 2:**
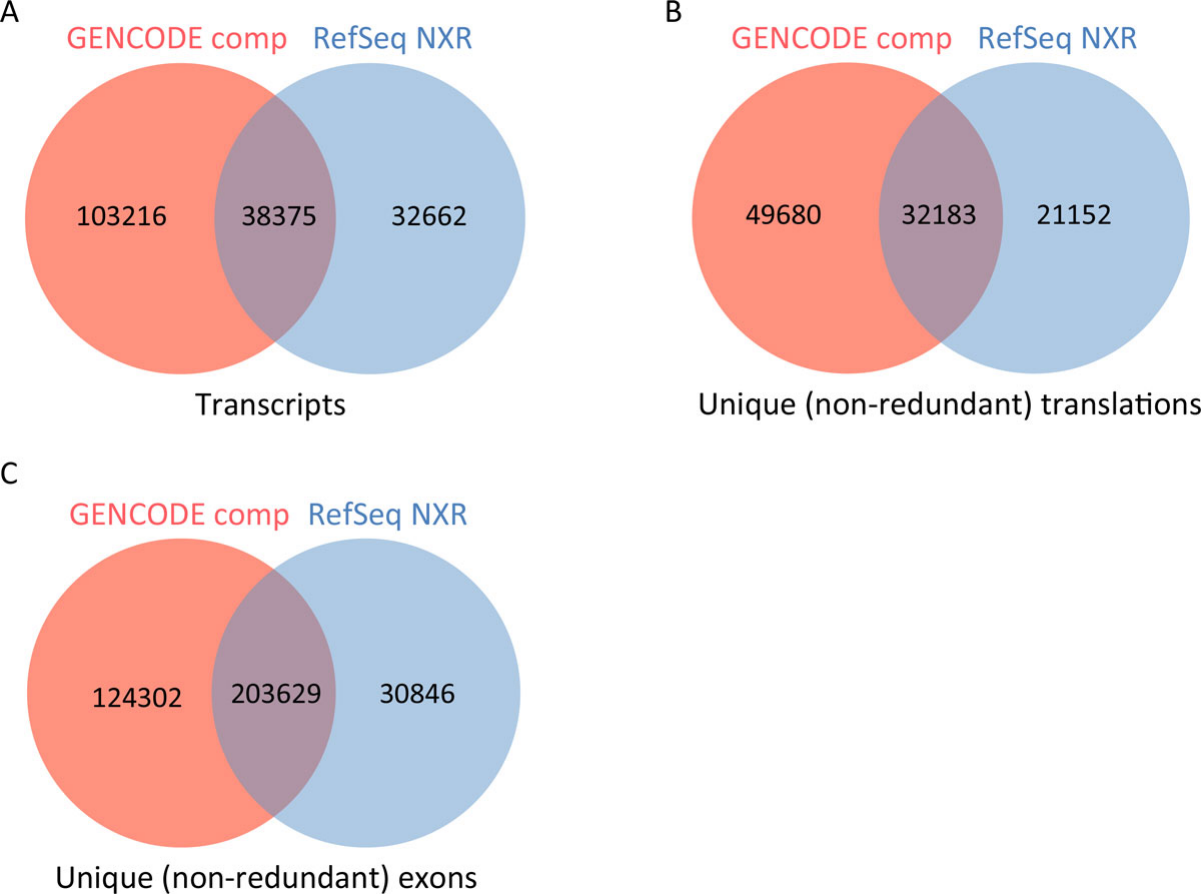
**Common and unique annotated features of GENCODE and RefSeq protein-coding genes**. Venn diagram to show intersection between A) transcripts annotated at GENCODE Comprehensive and RefSeq NXR protein-coding loci B) unique (non-redundant) translations annotated at GENCODE Comprehensive and RefSeq NXR protein-coding loci C) unique (non-redundant) exons annotated at GENCODE Comprehensive and RefSeq NXR protein-coding loci

The GENCODE Comprehensive geneset contains more than three times as many unique transcripts as RefSeq NXR (Figure 2A), while GENCODE Basic has approximately half the unique transcripts of RefSeq NXR (Additional file 3: Table S1). Unsurprisingly, a very similar pattern is seen for unique translations (Figure 2B). While GENCODE Comprehensive and RefSeq NXR share more than 32,000 translations, the former has greater than two fold more than RefSeq NXR. While the GENCODE Basic set still shares more than 32,000 translations with RefSeq NXR, it has ~7,700 fewer unique translations (Additional file 4: Table S2). Identification of unique exons makes an obvious contribution to the annotation of unique transcripts and translations in both the GENCODE and RefSeq genesets. GENCODE Comprehensive has approximately four fold more unique exons than RefSeq NXR (Figure 2C), predominantly associated with transcripts with annotated CDSs (Additional file 5: Table S3). The genomic coverage of unique exons in all four genesets, and the relative contribution of each transcript biotype to the genomic coverage of unique exons are also detailed in Supplementary Table 3. The GENCODE Basic set has nearly 20% fewer unique exons than RefSeq NXR. In summary, we find that GENCODE comprehensive captures a great many more novel transcriptional features than the RefSeq NXR set, while GENCODE Basic set is more similar to RefSeq NXR.

### Expression of GENCODE and RefSeq transcripts

**Figure 3:**
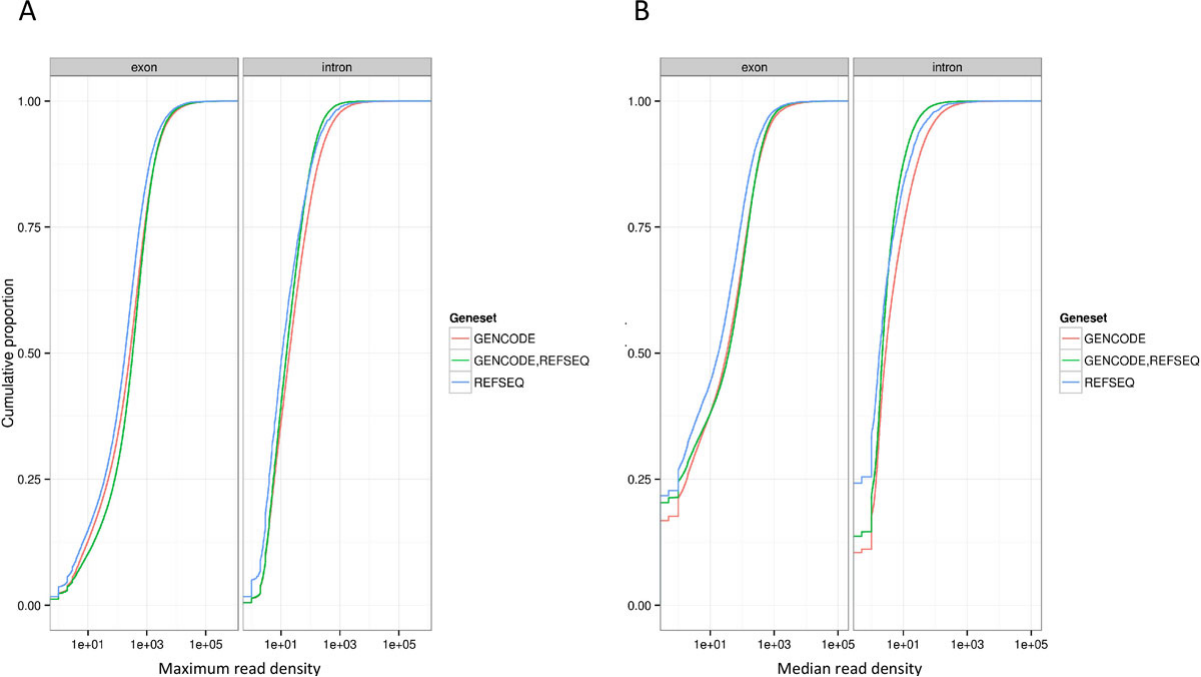
**Expression of GENCODE and RefSeq exons and introns**. Cumulative distibutions of RNAseq read count for GENCODE-only (Red), RefSeq-only (Blue) and GENCODE-RefSeq common (Green) exons and introns A) Shows maximum expression i.e. read density in the sample with highest expression B) Shows median expression i.e. read density level in the sample with median expresion

Using RNAseq data from 18 ENCODE cell lines, we investigated the expression of exons and introns belonging to protein-coding loci that were unique to either the GENCODE or RefSeq genesets or common to both. Mapping exon and intron expression data from GENCODE and RefSeq transcripts constructed on GRCh37 to the GRCh38-based transcripts shows that exons and introns in all three categories possess very similar expression characteristics, with their cumulative distributions mapping very closely to one another, particularly where maximum expression is considered (Figure 3A). A comparison of median expression across the 18 cell lines produces a slightly greater separation between the cumulative distributions. The higher y-intercept (for example 0.25 of all RefSeq-only introns vs 0.12 of all introns annotated by both GENCODE and RefSeq and 0.1 of all GENCODE only introns) indicates more features with a median of zero expression, and the small leftward-shift of the curve for median expression of exons highlights a slightly higher proportion of RefSeq-only exons, with lower expression than GENCODE-RefSeq common and GENCODE-only features (Figure 3B). These data indicate that the exons and introns common to both GENCODE and RefSeq genesets are expressed, and the features unique to both RefSeq and GENCODE are as robust and reliable as those held in common. This is particularly significant with regard to the GENCODE Comprehensive set, given that it has four times as many unique exons as the RefSeq NXR set.

### Impact of reference transcript set on variant annotation

To contrast the outcomes of using either the GENCODE or RefSeq genesets in the study of genome variation, we used the Ensembl VEP [6] to annotate variants from a genome and exome sequencing study (1KG) [1] and an exome-only sequencing study (ESP) [29], separately using the GENCODE and RefSeq genesets for transcript annotation. It is important to note that the exome library used for capture in the ESP study is based on RefSeq transcript annotation. Where variation maps to transcripts from both genesets we define the variant annotation as 'concordant'. For variation that does not fit these criteria, there are two ways in which variant annotation can diverge: (1) where a variant overlaps a transcript in both sets but is assigned an alternative functional consequence due to differing transcript annotation (we define as 'discordant' variant annotation), and (2) where a variant overlaps a transcript in one geneset but not the other (we define as 'unique' variant annotation).

Additional file 6: Figure S3 and Additional file 7: Figure S4 show the intersection between the GENCODE Comprehensive and Basic sets, and RefSeq NXR and NR sets, for 1KG and ESP variants respectively. Overall, the majority of variants map to transcripts in both genesets. GENCODE Comprehensive and RefSeq NXR share 68% of 1.36 million 1KG variants that map to at least one geneset, while 82% of the 1.1 million 1KG variants mapping to GENCODE Basic and RefSeq NXR are common to both sets. For the exome data, GENCODE Comprehensive shares 93% of 1.4 million ESP variants with RefSeq NXR, and GENCODE Basic and RefSeq NXR share 98% of 1.33 million ESP variants.

The number of discordant consequence calls for variants that map to both genesets was low for every comparison. For 1KG variants, 29,376 (3.1%) of variants in common had different calls when using GENCODE Comprehensive and RefSeq NXR as the reference gene annotation, compared with just 9,974 (1.1%) between GENCODE Basic and RefSeq NXR. For the ESP set, discordant calls were identified for 22,499 (1.7%) and 11,147 (0.9%) of variants respectively. The second, and larger source of difference between consequence predictions arises from variants that map to only one dataset. Additional file 8: Figure S5 shows that, for the 1KG variants, 404,145 variants map only to GENCODE Comprehensive transcripts and 84,464 map only to RefSeq NXR transcripts. There are also 121,107 variants that map only to GENCODE Basic transcripts compared to 80,999 mapping to RefSeq NXR transcripts. A similar pattern is present for the ESP setdata 84,265 variants map exclusively to GENCODE Comprehensive and 8,570 variants map only to RefSeq NXR. Conversely, 14,179 variants map only to RefSeq NXR while only 12,044 map only to GENCODE Basic.

The largest classes of variants in the 1KG dataset that are called concordantly when comparing GENCODE Comprehensive and GENCODE Basic with RefSeq NXR genesets have CDS and UTR and non-coding transcript consequences. Splice-site proximal variants and LoF variants are considerably less highly represented (Additional file 9: Figure S6 A and B). For ESP data, concordant variants are significantly more likely to have a consequence associated with a CDS than any of the other consequences, which are equally well represented (Additional file 9: Figure S6 C and D). For most datasets and variant consequences, concordant calls are higher than discordant and unique calls. The exceptions to this are UTR and non-coding transcript consequences for variants unique to the GENCODE Comprehensive set in both 1KG and ESP datasets and to a lesser extent GENCODE Basic and RefSeq NXR 'other' variants when compared using both the 1KG and ESP. A description of variant classification into the broad groups 'LoF', 'CDS', 'splice' and 'other' can be found in Additional file 10: Table S4. For both 1KG and ESP datasets, transcripts in the GENCODE Comprehensive geneset overlap with more variants in all broad groups of consequences than RefSeq NXR transcripts. The opposite is true for transcripts in the GENCODE Basic which overlap fewer variants than RefSeq NXR transcripts for variants in all broad groups of consequences except UTR and non-coding transcript, 'other' variants in the 1KG dataset.

The distribution of variant consequences is recapulated by looking at the porportion of each class of variants within the concordant, discordant and unique variant sets. CDS and 'other' variants compose approximately 50% of the concordant transcripts, in the 1KG dataset and ~85% in the ESP dataset. Discordant variants mapping to the GENCODE Basic and Comprehensive transcripts comprise 30-40% of CDS variants for the 1KG dataset and ~60% in the ESP dataset with a corresponding reduction in the 'other' variants (Additional file 11: Figure S7). In every case RefSeq NXR discordant variants follow the same pattern with a slightly higher proportion of CDS variants than discordant variants in GENCODE. For variants that only map to transcripts from one geneset, there is a much lower porportion of CDS variants and corresponding increase in 'other' variants, indeed the highest proportion of CDS variants mapping to transcripts from only one geneset is less than 40%, in the GENCODE Basic vs RefSeq NXR comparsion of the ESP dataset.

The proportion of discordant and unique LoF, missense and synonymous variants contributed by each geneset reaveal large differences dependent on the reference gene annotation used (Additional file 12: Figure S8). For both 1KG and ESP datasets, the GENCODE Comprehensive geneset contributes between 55-80% of all non-concordant LoF variants and missense variants, only synonymous variants show a different pattern with 60% being contributed by the RefSeq NXR geneset. For the GENCODE Basic geneset, the pattern is similarly consistent, but reversed with the RefSeq NXR contributing 60-65% of all non-concordant LoF, synonymous and missense variants.

**Figure 4:**
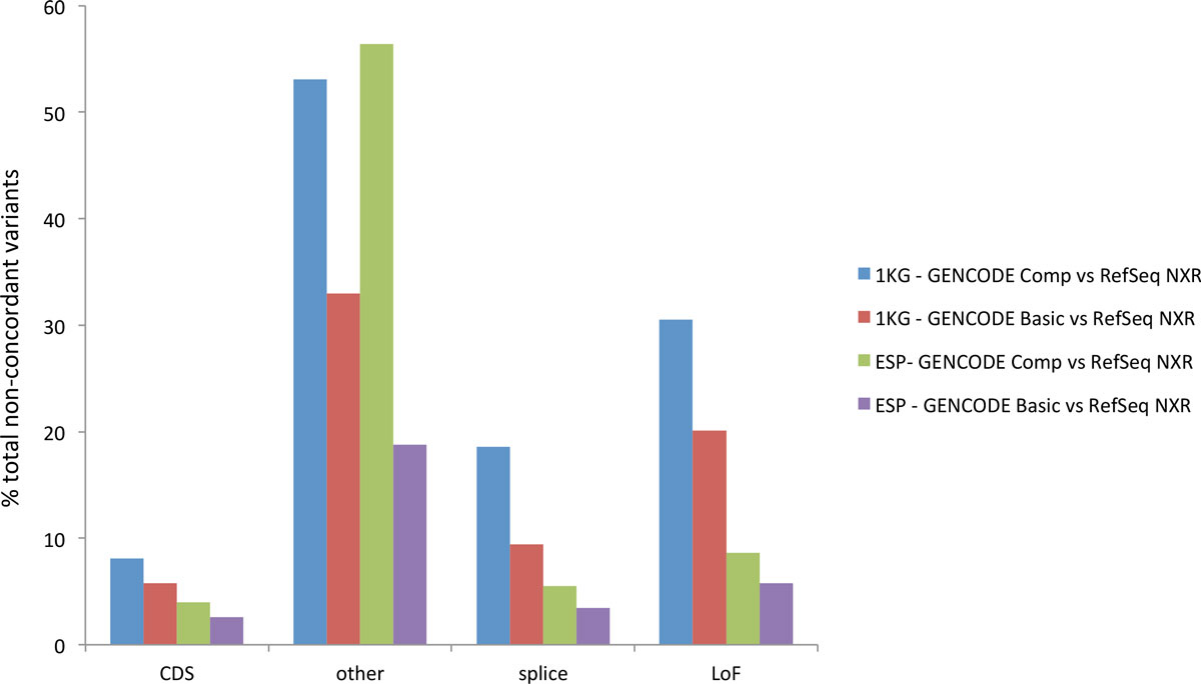
**Non-concordance of variant functional annotation**. Percentage non-concordant annotation i.e. variants with annotation in only one dataset (unique) or different annotation between datasets (discordant). The variants are represented in four broad classes; CDS, other, splice and LoF with comparisons between GENCODE Comprehensive and RefSeq NXR using 1KG data (Blue), GENCODE Basic and RefSeq NXR using 1KG data (Red), GENCODE Comprehensive and RefSeq NXR using ESP data (Green), and GENCODE Basic and RefSeq NXR using 1KG data (Purple).

Overall, variants affecting non-coding and UTR ('other') variants are the largest group in 1KG data, while CDS variants are the largest group in ESP data (Additional file 9: Figure S6 A/B and C/D respectively). The two datasets also represent the extremes of the concordance identified in variant annotation, with CDS variants showing high (>90%) concordance in all conditions while 'other' variants show high discordance (up to 56%). One of the most striking findings demonstrated by Macarthy et al. was that only 44% of LoF variants were identified in common in the two transcript sets [12]. Our own observation is that approximately 30% of LoF variant calls are in conflict (Figure 4). This difference may be due to the use of a different variant annotation tools (VEP vs Annovar), or the fact that both genesets may have changed substantially since the releases used in the earlier study. The most significant differences we identify are between GENCODE Comprehensive and RefSeq NXR, which is not surprising since they contain the most novel transcripts, splicing features and highest genomic coverage. Similarly, variation identified in the 1KG variant set shows considerably more variation than that from the ESP set, reflecting the additional genic features not captured by exome sequencing, and emphasising that exome design will inevitably lag behind transcript annotation.

## Discussion

It is clear that there are significant differences between the GENCODE and RefSeq genesets. The GENCODE Comprehensive set contains more AS, more novel CDSs, more novel exons and a higher genomic coverage than the full RefSeq annotation. This is despite the inclusion of RNAseq-based computationally-predicted 'XM' transcripts in the RefSeq geneset. One explanation for this is that the RefSeq AS complement seems enriched for exon-skipping or novel exon combinations, i.e. intronic features, neither of which increase genomic coverage. In contrast, transcripts in both the GENCODE Comprehensive and Basic sets have longer 5'and 3' UTRs, which contributes to the overall greater genomic coverage. Furthermore, the GENCODE comprehensive set includes two classes of transcripts that lack CDS: 'retained intron' transcripts, and those where the truncated nature of the supporting evidence makes the coding potential of the model ambiguous ('processed transcripts'). One consequence of the additional genomic coverage in GENCODE due to UTRs and non-coding transcripts is that much of the discordance in variation calling we observe is annotated as non-coding RNA or 5'/3' UTR-linked. That is not to say such variation is unimportant; UTR variation can affect many aspects of regulation (e.g. mRNA stability [30,31] and protein translation [32,33]) and while the sequences underlying these processes are largely cryptic at the present time, we predict they will be considered a more significant source of functional variation in future. Similarly, processed-transcripts (and RefSeq 'NR' transcripts) within protein-coding genes are in fact likely to encode CDS in reality, whether they are full-length or targets for the NMD pathway. It may thus be appropriate for certain variation studies to incorporate information regarding such putative CDSs, depending on the overall goals of the study. Even retained introns may not simply reflect the capture of immature transcripts or splicing aberrations, with several instances of functional intron retention being reported [34,35].

While relatively low, the discordance in CDS variant calling is likely to be problematic given the greater emphasis currently placed on the propensity of coding variation to be causal for phenotypic difference. For example, the identification of potentially deleterious missense mutations by the SIFT and PolyPhen2 components of the Ensembl VEP provides a clear starting point in the search for candidate disease-causing variants. However, differences between the genesets in terms of CDS length, reading frame or especially the presence or absence of the CDS could increase false positive reports, thus complicating interpretation. This captures the dichotomy at the heart of variant annotation. While one researcher might want to capture a large set of plausible functional variants, another may require the clarity of interpretation afforded by a reduced false positive rate. The GENCODE Comprehensive geneset includes more splicing features than GENCODE Basic, and it covers more genomic sequence. RNAseq data supports these additional exons and introns being expressed at least as highly as those features shared by GENCODE and RefSeq. GENCODE Comprehensive also captures more LoF, coding and splice region variants than the most complete RefSeq set. In contrast, GENCODE Basic is a less complex geneset, containing fewer full-length protein-coding models. As a consequence, GENCODE Basic shows less discordant variant annotation, and captures fewer unique LoF, coding and splice region variants than the most complete RefSeq set. Analysis of dominant transcript expression indicates that the GENCODE Basic set is enriched for highly expressed transcripts (see Additional file 13: Dominant expression analysis). Unfortunately, transcript reconstruction and quantification from RNAseq is not sufficiently reliable to allow tissue-specific filtering of transcripts on the basis of expression at present, but it does permit the most highly expressed transcripts to be identified with reasonable confidence. This will provide a useful basis on which to simplify the transcript set, particularly in combination with principal isoform call from APPRIS which is also inlcuded in GENCODE.

## Conclusions

GENCODE has a higher proportion of manually annotated gene models than RefSeq and includes more novel splicing features. Given our modern understanding of 'pervasive transcription', one could question to what extent this excess transcription is truly functional, as opposed to potential 'noise'. We have demonstrated that the novel exons and introns annotated by GENCODE and RefSeq share characteristics of transcription with those features already annotated in both sets, suggesting that transcriptional noise is unlikely to be the major explanation for the existence of such transcripts, or at least no more so than for transcripts already independently added to both genesets. The additional coverage and diversity of GENCODE Comprehensive transcripts leads to the identification of many more genic variants than RefSeq, however, transcriptional complexity can also make variant interpretation more difficult (see Additional file 14: Figure S9). The GENCODE Basic geneset shares may characteristics with RefSeq, although it captures fewer novel LoF and coding variants. Furthermore, while transcript level quantification is not currently sufficiently reliable to be used as a basis for filtering transcripts in a tissue-specific manner, simply asking which is the dominantly expressed transcript holds some promise, and the GENCODE Basic set, contains the vast majority of transcripts identified as dominant. This suggests it represents an effective filter for functional transcripts, in lieu of more reliable transcript quantification becoming available from the use of longer read technologies.

## Methods

### GENCODE gene annotation

Manual annotation of protein-coding, long non-coding RNA and pseudogene loci was undertaken using the guidelines of the HAVANA (Human And Vertebrate Analysis and Annotation) group; which can be found at ftp://ftp.sanger.ac.uk/pub/annotation. The manual annotation of protein-coding loci is predominantly created based on support from the alignment of transcriptomic (ESTs and mRNAs) and proteomic data from GenBank and Uniprot. Ensembl annotation of protein-coding genes is accomplished using an automated pipeline. [19] Protein sequences from UniProt [36] were included as input, along with RefSeq sequences. Untranslated regions (UTRs) were added using cDNA sequences from the EMBL Nucleotide Archive (ENA) [37].

The final GENCODE geneset is the result of merging the HAVANA and Ensembl annotation. During the merge process, all HAVANA and Ensembl transcript models are compared, by clustering transcripts with overlapping exons containing a CDS on the same strand, followed by pairwise comparisons of all exons in a transcript cluster. Prior to this manual annotation is subject to strict QC and any highlighted transcripts are referred back to HAVANA for reinspection. A more detailed description is reported in Harrow et al. [14]

### Comparison of GENCODE and RefSeq gene and transcript annotation

The datasets used for comparative analysis were GENCODE v21 (obtained from the homo_sapiens_core_77_38 database) and RefSeq (NCBI Homo sapiens Annotation Release 106 as imported in Ensembl 77 (homo_sapiens_otherfeatures_77_38 database, 'RefSeq_import' analysis)). Only gene annotation on the main chromosomes of GRCh38 were included, i.e. genome patches, alternative alleles and the mitochondrial genome were excluded. All transcripts from GENCODE genes with the locus biotype 'coding' (i.e. protein-coding) were included; all genes with locus biotypes 'lncRNA', 'pseudogene', 'IG' or 'TR' were excluded. All transcripts from RefSeq genes with the locus biotype 'coding' were included alongside any transcripts from loci with the biotype 'misc_RNA', where any transcript from that locus possessed a CDS. Thus transcripts from loci with the biotypes lncRNA and pseudogene were excluded, along with any transcripts belonging to loci with biotype 'misc_RNA' where no transcript at the locus possessed a CDS. The genesets were defined as follows; GENCODE Comprehensive contains all transcripts at protein-coding loci, GENCODE Basic contains only transcripts tagged as 'basic' i.e. only protein-coding transcripts (not including NMD transcripts) with a full-length CDS with start and stop codon identified. This excludes any truncated transcripts with CDS_start_NF ('Not Found') and CDS_end_NF tag, and any transcripts with transcript biotype 'NMD', 'retained_intron', 'processed_transcript'. RefSeq NXR contains all transcripts, known (with NM or NR prefix) or predicted (XM, XR), in genes containing at least one known transcript, and RefSeq NR contains only known RefSeq transcripts (NM or NR).

In order to calculate the number of transcripts and translations held in common or unique to each geneset we compared, for each transcript in every pair of geneset: (1) the exon coordinates in the case of single-exon transcripts, (2) the intron coordinates in the case of multi-exon transcripts (in order to compensate for different UTR lengths), and (3) the CDS exon coordinates in the case of translations. Unique exons were defined as having at least one unique splice site; all exons that are first or last exons of a transcript were excluded them from the set if their splice junction was shared with another, longer exon, which was retained in the set. Where internal exons overlap but share different splice junctions, they were called as unique and retained in the set; where splice junctions were shared with another exon then only one copy of the exon was retained for the calculation of coverage. While some genome sequence may be redundant e.g. where two exons shared a common splice donor site but had different splice acceptors, the set is non-redundant at the exon and transcript level, e.g. where two exons shared the same splice donor and acceptor, or for terminal exons that shared one splice junction but differed in length. In such cases only one copy was retained in the set. Genomic coverage of unique exons was calculated by summing all the unique exon lengths, separately for each strand.

### Analysis of exon and intron expression in GENCODE and RefSeq

Two sources of transcript models were used; GENCODE v19 (http://www.GENCODEgenes.org/), RefSeq v19 (http://hgdownload.soe.ucsc.edu/goldenPath/hg19/database/). The list of RNA-seq samples and their respective GEO accession numbers are described here (http://www.biorxiv.org/content/early/2014/10/30/010884). Exons and introns were assigned into classes corresponding to the above sources or to a combination of them. An exon was said to be terminal if it was the first or the last exon in at least one transcript. The expression level for each exon and intron was computed by averaging the read density over the nucleotide span using the bigwigaverageoverbed utility. The expression level of an exon or intron was assessed by taking (a) the average and (b) the maximum read density across samples. We then projected this analysis onto the protein-coding genes in GENCODE Comprehensive release 21 and RefSeq NXR. The exon and intron comparisons have been made by projecting co-ordinates from GRCh37 (h19) to GRCh38. Again the exon sets are redundant i.e. if the same exons appear in multiple transcripts they will be counted multiple times. Exons that were added between release 19 and release 21 are not included in this analysis.

### Analysis of variant annotation with GENCODE and RefSeq

Two variant datasets were used for this analysis. Dataset 1 (1KG) contains variants from the EUR super-population (379 individuals) from the phase 1 release of the 1000 Genomes Project [1]. This includes data from both low coverage whole-genome sequencing and high coverage exome sequencing. The exome capture is detailed here (http://www.1000genomes.org/category/exome). Dataset 2 (ESP) contains variants from the European-American population (4,298 individuals) from the final release of the ESP data (ESP6500) [29]. Exome capture was performed using the Nimblegen SeqCap EZ v2, which was designed against RefSeq (Jan 2010), CCDS (Sept 2009), and miRBase (Sept 2009). Variants were mapped to GRCh38 by Ensembl (release 76). All variation data used can be accessed here (ftp://ftp.ensembl.org/pub/release-76/variation/vcf/homo_sapiens/)

Variant annotation was performed using Ensembl/VEP version 76 (August 2014 release) with standard parameters, -- RefSeq to use the Ensembl mapping of RefSeq transcripts, -- GENCODE_basic to limit to transcripts in the GENCODE Basic set. Custom scripts (also based on Ensembl release 76) were used to filter the annotations to only include annotations from protein-coding loci (defined as those with at least one transcript in the gene having a biotype of 'protein_coding') and to variants annotated as falling in an exon or the proximal splice region. For some analyses a single consequence call was selected for each variant according to the 'severity' ranking used by Ensembl and identified in the table here (http://aug2014.archive.ensembl.org/info/genome/variation/predicted_data.html).

## Competing interests

No competeing interests

## Authors' contributions

AF, JMM and JH conceived and designed the study. AF coordinated analysis and writing. JMG and AF performed dataset comparison. DP and BU performed expression analysis. RP, BU and NF performed dominant transcript analysis. GRSR and AF performed variant annotation analysis. AF, JMG, JMM, DP, RP, GRSR, BU, AB, RG, and JH helped to draft the manuscript. All authors read and approved the final manuscript.

## Supplementary Material

Additional file 1 **Figure S1 - Comparison of GENCODE and RefSeq Annotation in the Ensembl genome browser**. Screenshot of the Ensembl genome browser displying the BRCA1 locus. GENCODE gene annotation is shown at the top of the panel. Manually annoated protein-coding transcripts are shown in red, manually annotated NMD, processed_transcript and retained_intron transcripts are shown in royal blue and merged manual and computational transcripts are shown in gold. RefSeq transcripts are shown in dark blue. Computationally predicted XM transcripts are highlighted by blue arrowheads, manually annotated, protein-coding NM transcripts in green and manually annotated non-coding NR transcripts in red. Click here for file

Additional file 2 **Figure S2 - Comparison of GENCODE and RefSeq Annotation in the UCSC genome browser**. Screenshot of the UCSC genome browser displying the SLC25A17 locus. RefSeq gene annotation is shown in dark blue in the top panel. GENCODE gene annotation is shown in the middle panel in blue (protein-coding and NMD transcripts) and green (processed_transcripts). CCDS transcripts are shown in the lowest panel in green. Novel GENCODE splicing features are highlighted in red (novel cassette exons), blue (novel alternative splice site, or shifted splice site) and green (novel putative TSS and 5' UTR). Click here for file

Additional file 3 **Table S1 - Intersection of transcripts in GENCODE and RefSeq annotation**. Number and functional biotypes of all transcripts shared by both genesets and unique to one in pairwise comparisons of all combinations of GENCODE Comprehensive, GENCODE Basic, RefSeq NXR and RefSeq NR (excluding the subsets GENCODE Comprehensive vs Basic and RefSeq NXR vs NR). Click here for file

Additional file 4 **Table S2 - Intersection of translations in GENCODE and RefSeq annotation**. Number of translations shared by both genesets and unique to one in pairwise comparisons of all combinations of GENCODE Comprehensive, GENCODE Basic, RefSeq NXR and RefSeq NR. Click here for file

Additional file 5 **Table S3 - Intersection of exons in GENCODE and RefSeq annotation**. Number, transcript functional biotype and genomic coverage of all exons shared by both genesets and unique to one in pairwise comparisons of all combinations of GENCODE Comprehensive, GENCODE Basic, RefSeq NXR and RefSeq NR. Click here for file

Additional file 6 **Figure S3 - Intersection of 1KG variants with four genesets**. Four-way Venn diagram to show the intersection of 1KG variants with GENCODE Comprehensive, GENCODE Basic, RefSeq NXR and RefSeq NR genesets. Click here for file

Additional file 7 **Figure S4 - Intersection of ESP variants with four genesets**. Four-way Venn diagram to show the intersection of ESP variants with GENCODE Comprehensive, GENCODE Basic, RefSeq NXR and RefSeq NR genesets. Click here for file

Additional file 8 **Figure S5 - Absolute values of concordance in the functional annotation of variation**. Numbers of concordant, discordant and unique variants. Concordant indicates variant given same annotation in both sets, discordant indicates the variant is found in both sets but given different annotation, and unique indicates variant is given functional annotation in only one set. Numbers for Gencode Comprehensive (GC), Gencode Basic (GB) and RefSeq NXR (NXR), for 1 KG data A) and ESP data B). Click here for file

Additional file 9 **Figure S6 - Numbers of concordant, discordant and unique variants by broad functional class** ('CDS', 'other', 'splice', 'LoF') in a pair consisting of either GENCODE Comprehensive (Gencode comp) and RefSeq NXR or GENCODE Basic and RefSeq NXR for 1KG variants A) and B) for ESP variants C) and D). Click here for file

Additional file 10 **Table S4 - Derivation of broad variant classes**. Derivation of grouping of broad variant classes from functional annotation terms from VEP. Click here for file

Additional file 11 **Figure S7 - Percentage of variant annotation by broad functional class** ('CDS, 'other', 'splice', 'LoF') of concordant, discordant and unique variants in a pair consisting of either GENCODE Comprehensive (Gencode comp) and RefSeq NXR or GENCODE Basic and RefSeq NXR for 1KG variants A) and B) for ESP variants C) and D). Click here for file

Additional file 12 **Figure S8 - Proportion of discordant and unique LoF and coding variants by variant consequence**. Percentage of discordant and unique variant annotation for specific annotations of variants in the broad LoF class and coding synonymous and missense coding variants in a pair consisting of either GENCODE Comprehensive (GC) and RefSeq NXR (NXR) or GENCODE Basic (GB) and RefSeq NXR (NXR) for 1KG variants A) and B) for ESP variants C) and D). Click here for file

Additional file 13 **Dominant expression analysis**. Results and methods for the analysis of dominantly expressed GENCODE transcripts. Click here for file

Additional file 14 **Figure S9 - Number of predicted consequences per variant**. Box plot of the number of predicted consequences for each variant by geneset. Click here for file

Additional file 15 **Figure S10 - Comparison of dominant transcript calls between FluxCapacitor and Cufflinks2**. Percentage of agreement between dominant transcripts assigned by FluxCapacitor and Cufflinks2 at all protein genes across 154 ENCODE 2 cell lines. Click here for file

Additional file 16 **igure S11 - Comparison of dominant transcript calls from FluxCapacitor and Cufflinks2 with APPRIS pipeline**. Percentage of agreement between dominant transcripts assigned by FluxCapacitor and Cufflinks2 and APPRIS principal isoforms at all protein genes across 154 ENCODE 2 cell lines. Dominant transcripts reported by Cufflinks2(Grey bars) and FluxCapacitor(Blue) are shown. Click here for file

Additional file 17 **Figure S12 - Comparison of dominant transcript calls from FluxCapacitor and Cufflinks2 with GENCODE Basic geneset**. Percentage of agreement between dominant transcripts assigned by FluxCapacitor and Cufflinks2 and GENCODE Basic transcripts at all protein genes across 154 ENCODE 2 cell lines. Dominant transcripts reported by Cufflinks2(Grey bars) and FluxCapacitor(Blue) are shown. Click here for file
