# Supplementary material for: Comparison of GENCODE and RefSeq gene annotation and the impact of reference geneset on variant effect prediction
Source: BMC Genomics. 2015 Jun 18;16(Suppl 8):S2. doi: 10.1186/1471-2164-16-S8-S2 (PMC4502323; doi:10.1186/1471-2164-16-S8-S2)
Supplement: Additional file 3 — Table S1 - Intersection of transcripts in GENCODE and RefSeq annotation. Number and functional biotypes of all transcripts shared by both genesets and unique to one in pairwise comparisons of all combinations of GENCODE Comprehensive, GENCODE Basic, RefSeq NXR and RefSeq NR (excluding the subsets GENCODE Comprehensive vs Basic and RefSeq NXR vs NR). [file 1471-2164-16-S8-S2-S3.pdf]

Dataset1_vs_dataset2	TRANSCRIPTS				
	Unique to 1	Common	Unique to 2	Unique to 1 (by biotype)	Unique to 2 (by biotype)
GENCODE Comprehensive vs GENCODE Basic	87568	54023	0	retained_intron:25482<br>protein_coding:24678<br>processed_transcript:24499<br>nonsense_mediated_decay:12804<br>non_stop_decay:69 other:139	-
GENCODE Comprehensive vs RefSeq NXR	103216	38375	32663	protein_coding:42675<br>retained_intron:25246<br>processed_transcript:23852<br>nonsense_mediated_decay:11397<br>non_stop_decay:64 other:123	protein_coding:29879<br>misc_RNA:2785
GENCODE Comprehensive vs RefSeq NR	106811	34780	4023	protein_coding:45464<br>retained_intron:25360<br>processed_transcript:24070<br>nonsense_mediated_decay:11874<br>non_stop_decay:69 other:125	protein_coding:3280<br>misc_RNA:744
GENCODE Basic vs RefSeq NXR	18949	35074	35964	protein_coding:18755<br>nonsense_mediated_decay:194	protein_coding:31752<br>misc_RNA:4214
GENCODE Basic vs RefSeq NR	21372	32651	6152	protein_coding:21178<br>nonsense_mediated_decay:194	protein_coding:4224<br>misc_RNA:1929
RefSeq NXR vs RefSeq NR	32235	38803	0	protein_coding:29930<br>misc_RNA:2306	-
