# Supplementary material for: Comparison of GENCODE and RefSeq gene annotation and the impact of reference geneset on variant effect prediction
Source: BMC Genomics. 2015 Jun 18;16(Suppl 8):S2. doi: 10.1186/1471-2164-16-S8-S2 (PMC4502323; doi:10.1186/1471-2164-16-S8-S2)
Supplement: Additional file 4 — Table S2 - Intersection of translations in GENCODE and RefSeq annotation. Number of translations shared by both genesets and unique to one in pairwise comparisons of all combinations of GENCODE Comprehensive, GENCODE Basic, RefSeq NXR and RefSeq NR. [file 1471-2164-16-S8-S2-S4.pdf]

Dataset1_vs_dataset2	TRANSLATIONS		
	Unique to 1	Common	Unique to 2
GENCODE Comprehensive vs GENCODE Basic	36186	45677	0
GENCODE Comprehensive vs RefSeq NXR	49680	32183	21152
GENCODE Comprehensive vs RefSeq NR	51610	30253	2059
GENCODE Basic vs RefSeq NXR	13635	32042	21293
GENCODE Basic vs RefSeq NR	15484	30193	2119
RefSeq NXR vs RefSeq NR	21023	32312	0
