# Supplementary material for: Comparison of GENCODE and RefSeq gene annotation and the impact of reference geneset on variant effect prediction
Source: BMC Genomics. 2015 Jun 18;16(Suppl 8):S2. doi: 10.1186/1471-2164-16-S8-S2 (PMC4502323; doi:10.1186/1471-2164-16-S8-S2)
Supplement: Additional file 5 — Table S3 - Intersection of exons in GENCODE and RefSeq annotation. Number, transcript functional biotype and genomic coverage of all exons shared by both genesets and unique to one in pairwise comparisons of all combinations of GENCODE Comprehensive, GENCODE Basic, RefSeq NXR and RefSeq NR. [file 1471-2164-16-S8-S2-S5.pdf]

Dataset1_vs_dataset2	EXONS									
	Unique to 1	Unique to 1 (by biotype)	Coverage	Coverage percentage	Coverage percentage (by biotype)	Unique to 2	Unique to 2 (by biotype)	Coverage	Coverage percentage	Coverage percentage (by biotype)
GENCODE Comprehensive vs GENCODE Basic	101288	retained_intron:32411<br>processed_transcript:30830<br>protein_coding:23617<br>nonsense_mediated_decay:14135<br>other:285	36975789	0.5986	retained_intron:0.3099<br>processed_transcript:0.1370<br>protein_coding:0.0839<br>nonsense_mediated_decay:0.0637<br>other:0.004	0	-	0	0	-
GENCODE Comprehensive vs RefSeq NXR	124302	protein_coding:53308<br>retained_intron:31039<br>processed_transcript:28058<br>nonsense_mediated_decay:11689<br>other:208	58337572	0.9445	protein_coding:0.4653<br>retained_intron:0.2987<br>processed_transcript:0.1252<br>nonsense_mediated_decay:0.0520<br>other:0.0034	30846	protein_coding:28944<br>misc_RNA:1902	21186023	0.343	protein_coding:0.3249<br>misc_RNA:0.0181
GENCODE Comprehensive vs RefSeq NR	138377	protein_coding:61942<br>retained_intron:32306<br>processed_transcript:30521<br>nonsense_mediated_decay:13381<br>other:227	63039492	1.0206	protein_coding:0.5178<br>retained_intron:0.3067<br>processed_transcript:0.1338<br>nonsense_mediated_decay:0.0589<br>other:0.0034	12750	protein_coding:12200<br>misc_RNA:550	12560858	0.2034	protein_coding:0.1991<br>misc_RNA:0.0043
GENCODE Basic vs RefSeq NXR	33978	protein_coding:33588<br>nonsense_mediated_decay:390	24524382	0.3971	protein_coding:0.3951<br>nonsense_mediated_decay:0.0019	41348	protein_coding:37684<br>misc_RNA:3664	24227994	0.3923	protein_coding:0.3655<br>misc_RNA:0.0268
GENCODE Basic vs RefSeq NR	39071	protein_coding:38663<br>nonsense_mediated_decay:408	27082977	0.4385	protein_coding:0.4365<br>nonsense_mediated_decay:0.0020	16216	protein_coding:14538<br>misc_RNA:1678	13869893	0.2246	protein_coding:0.2160<br>misc_RNA:0.0085
RefSeq NX vs RefSeq NR	28764	protein_coding:26512<br>misc_RNA:2252	12219927	0.1978	protein_coding:0.1786<br>misc_RNA:0.0192	0	-	0	0	-
