# Supplementary figures and images for: Comparison of GENCODE and RefSeq gene annotation and the impact of reference geneset on variant effect prediction
Source: BMC Genomics. 2015 Jun 18;16(Suppl 8):S2. doi: 10.1186/1471-2164-16-S8-S2 (PMC4502323; doi:10.1186/1471-2164-16-S8-S2)

exonic variants 1KG

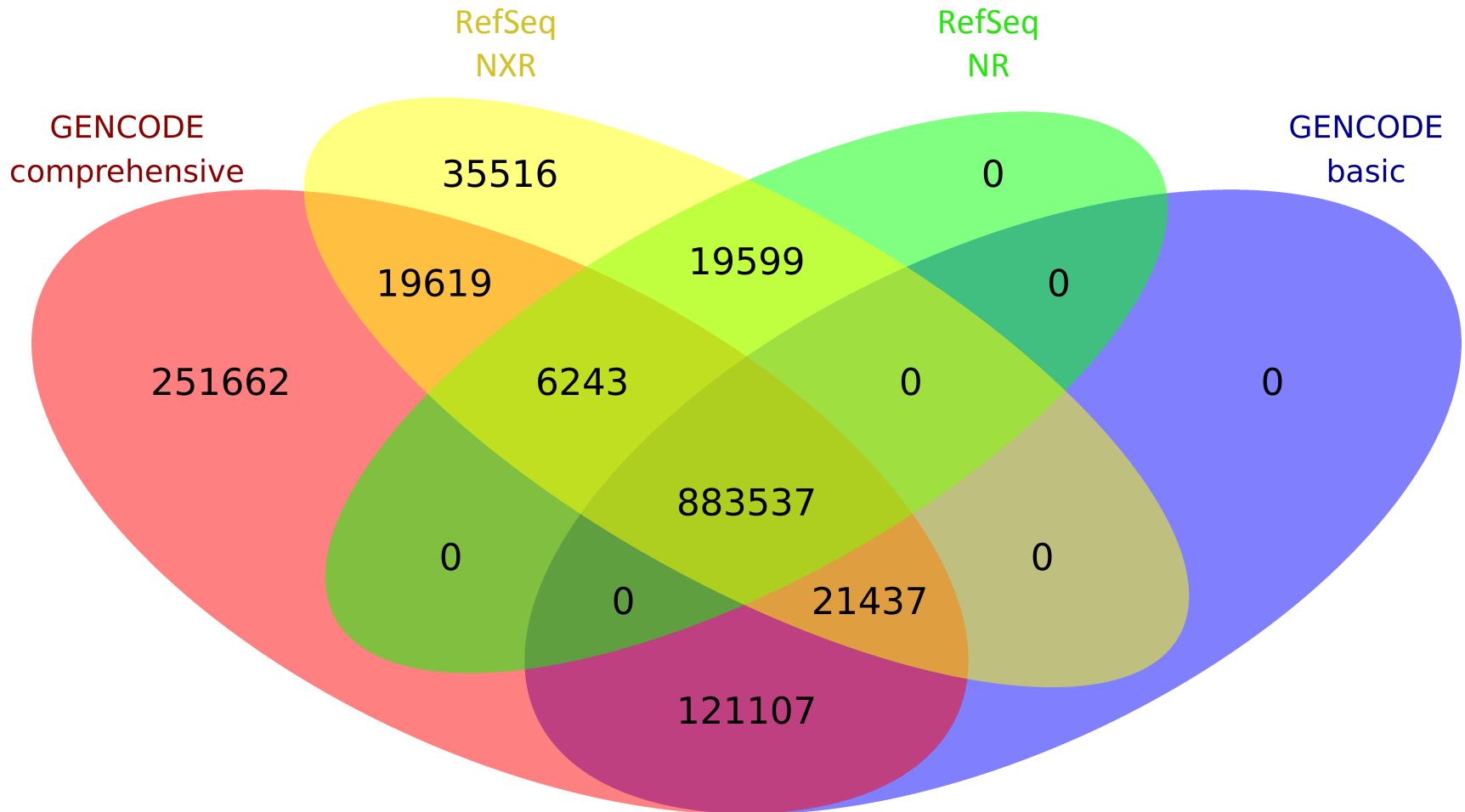

Supplement: Additional file 6 — Figure S3 - Intersection of 1KG variants with four genesets. Four-way Venn diagram to show the intersection of 1KG variants with GENCODE Comprehensive, GENCODE Basic, RefSeq NXR and RefSeq NR genesets. [file 1471-2164-16-S8-S2-S6.pdf]

exonic variants ESP

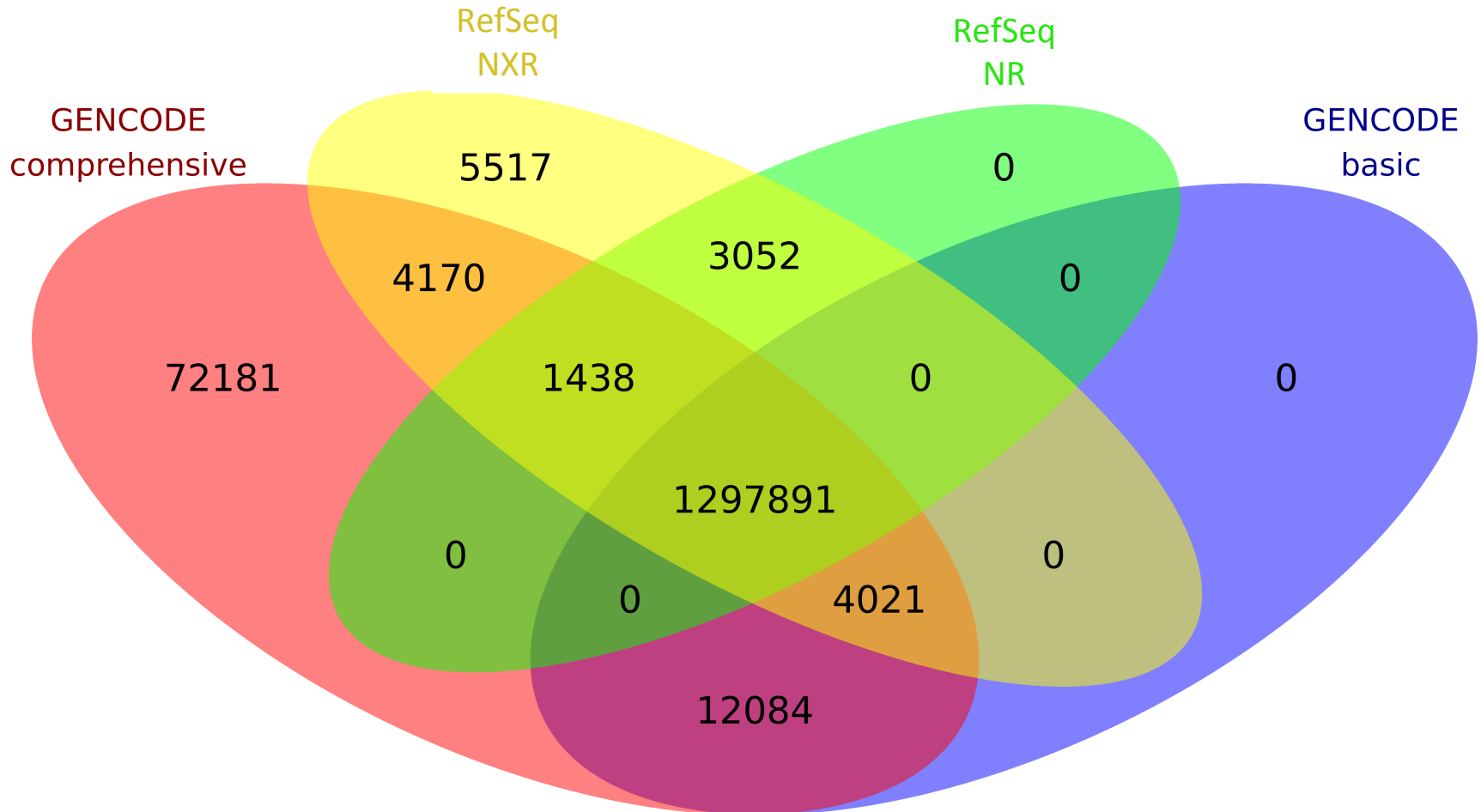

Supplement: Additional file 7 — Figure S4 - Intersection of ESP variants with four genesets. Four-way Venn diagram to show the intersection of ESP variants with GENCODE Comprehensive, GENCODE Basic, RefSeq NXR and RefSeq NR genesets. [file 1471-2164-16-S8-S2-S7.pdf]

A

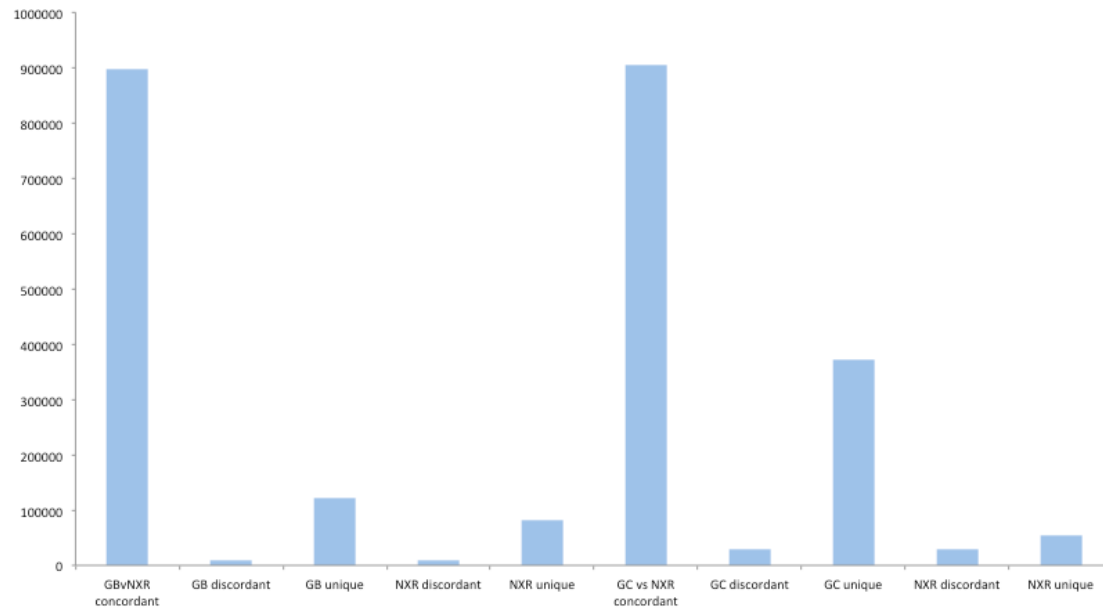

B

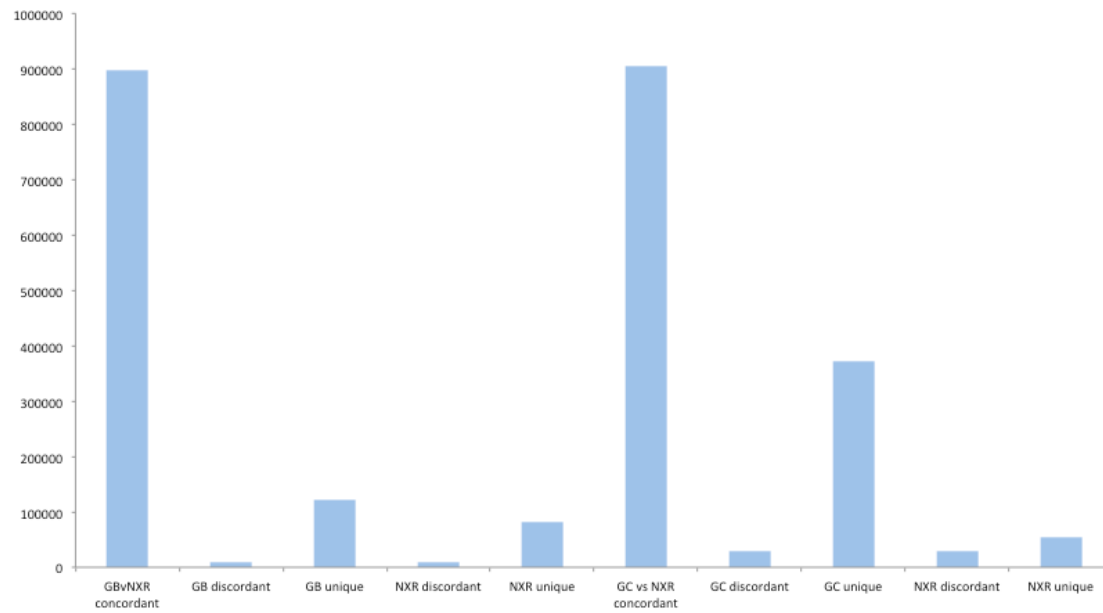

Supplement: Additional file 8 — Figure S5 - Absolute values of concordance in the functional annotation of variation. Numbers of concordant, discordant and unique variants. Concordant indicates variant given same annotation in both sets, discordant indicates the variant is found in both sets but given different annotation, and unique indicates variant is given functional annotation in only one set. Numbers for Gencode Comprehensive (GC), Gencode Basic (GB) and RefSeq NXR (NXR), for 1 KG data A) and ESP data B). [file 1471-2164-16-S8-S2-S8.pdf]

A

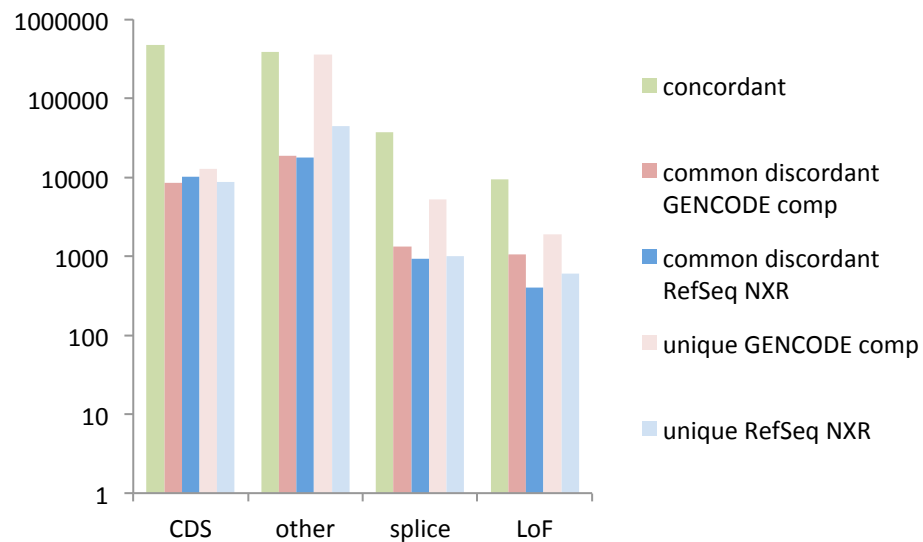

B

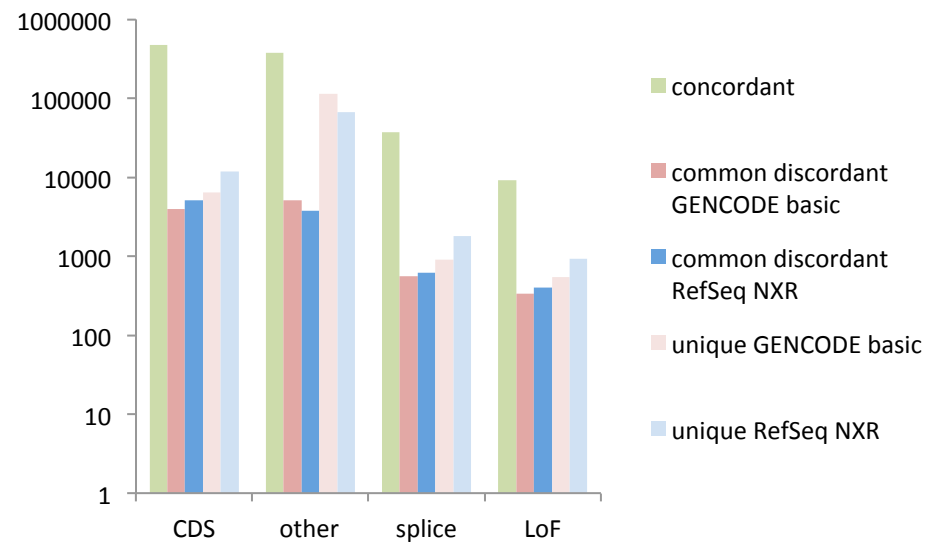

C

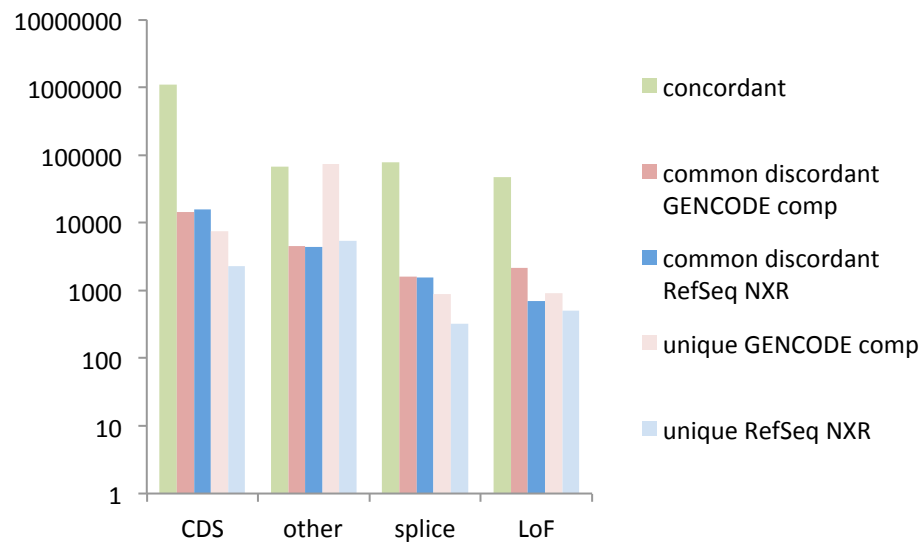

D

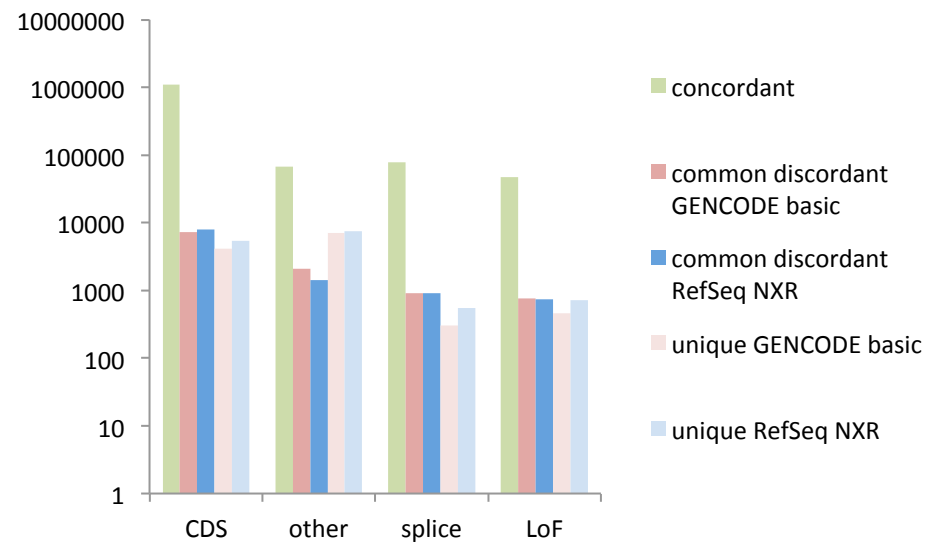

Supplement: Additional file 9 — Figure S6 - Numbers of concordant, discordant and unique variants by broad functional class ('CDS', 'other', 'splice', 'LoF') in a pair consisting of either GENCODE Comprehensive (Gencode comp) and RefSeq NXR or GENCODE Basic and RefSeq NXR for 1KG variants A) and B) for ESP variants C) and D). [file 1471-2164-16-S8-S2-S9.pdf]

A

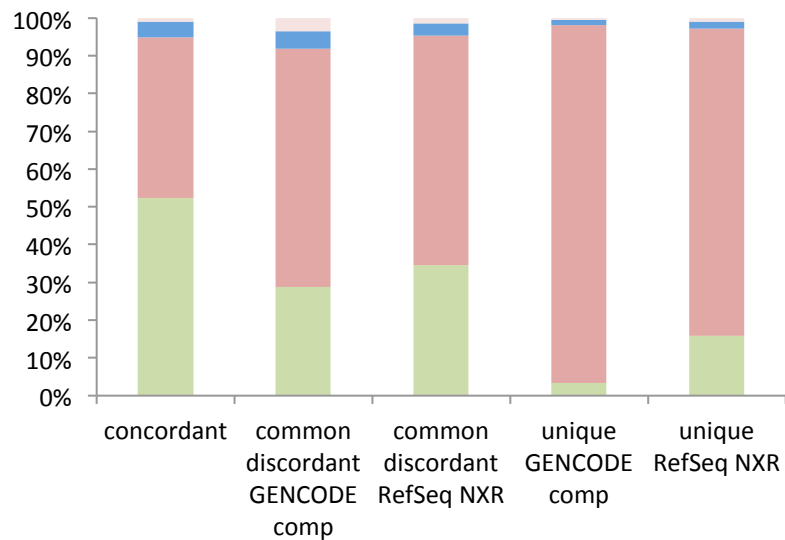

B

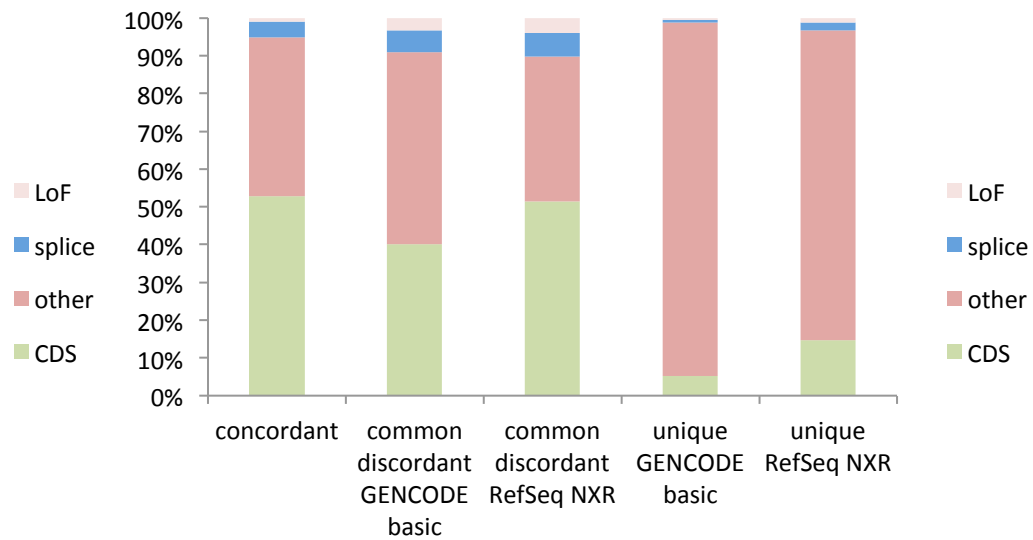

C

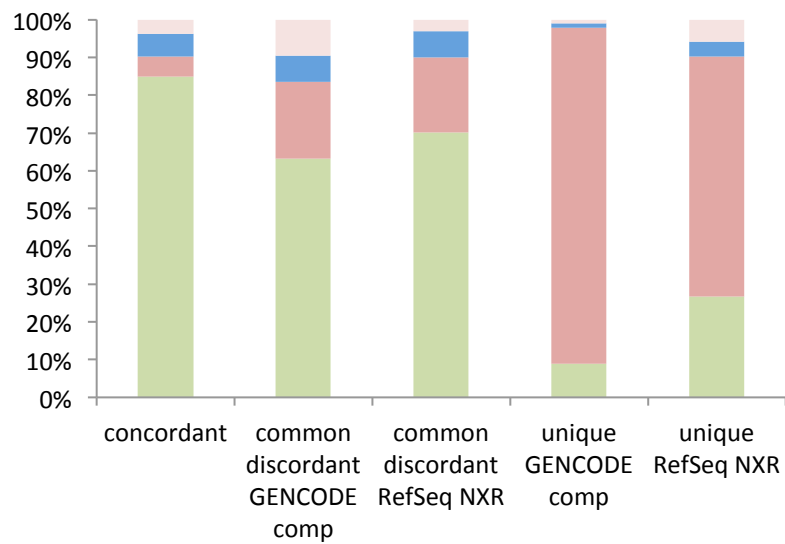

D

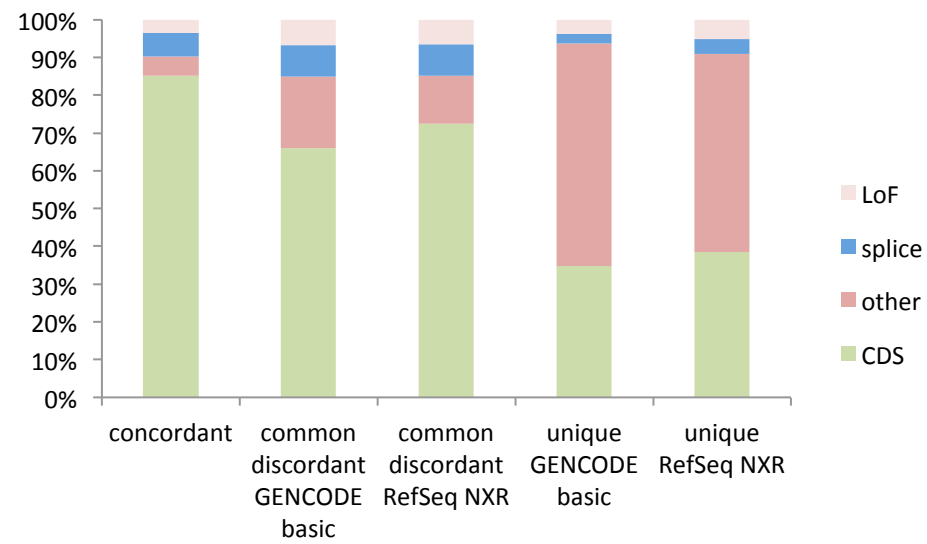

Supplement: Additional file 11 — Figure S7 - Percentage of variant annotation by broad functional class ('CDS, 'other', 'splice', 'LoF') of concordant, discordant and unique variants in a pair consisting of either GENCODE Comprehensive (Gencode comp) and RefSeq NXR or GENCODE Basic and RefSeq NXR for 1KG variants A) and B) for ESP variants C) and D). [file 1471-2164-16-S8-S2-S11.pdf]

A

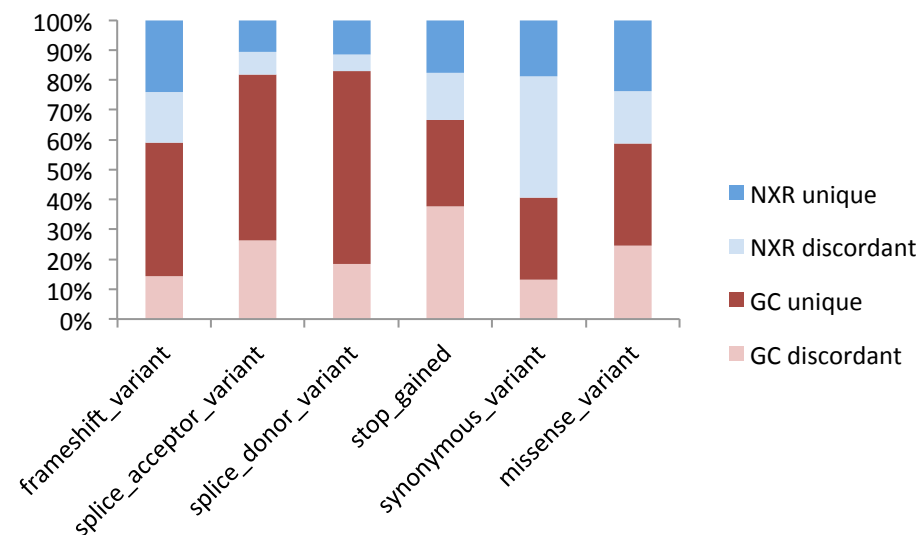

B

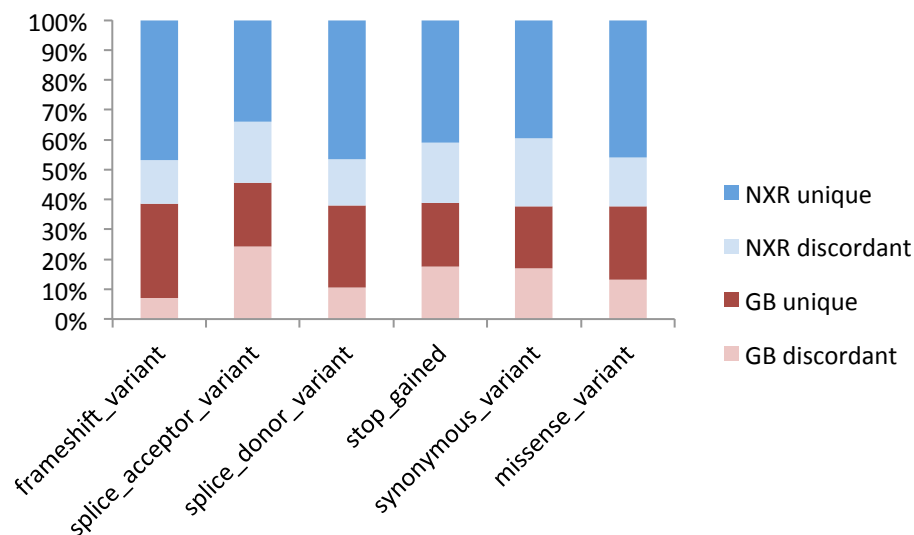

C

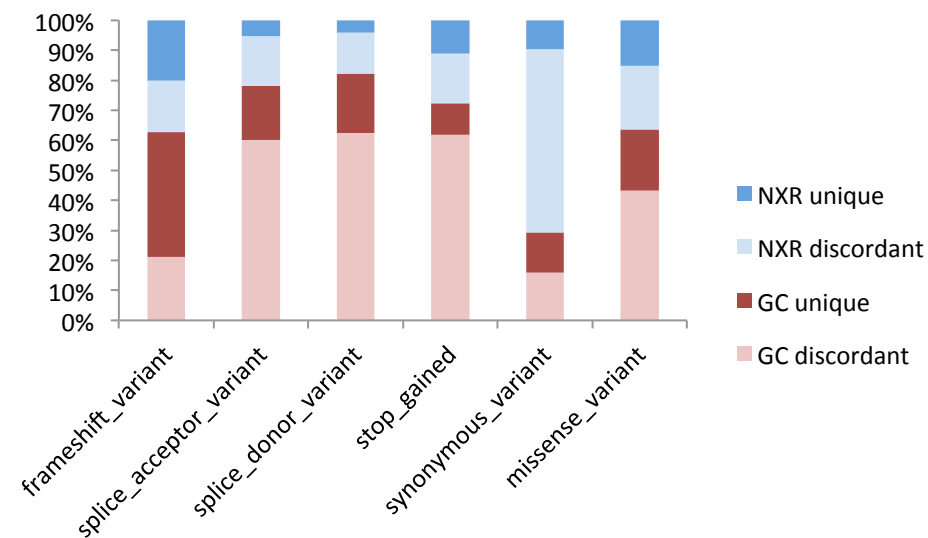

D

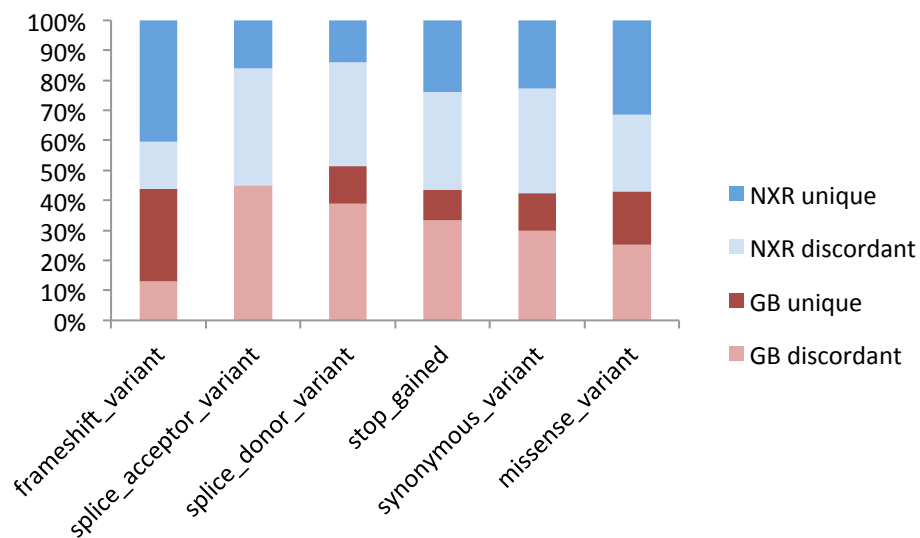

Supplement: Additional file 12 — Figure S8 - Proportion of discordant and unique LoF and coding variants by variant consequence. Percentage of discordant and unique variant annotation for specific annotations of variants in the broad LoF class and coding synonymous and missense coding variants in a pair consisting of either GENCODE Comprehensive (GC) and RefSeq NXR (NXR) or GENCODE Basic (GB) and RefSeq NXR (NXR) for 1KG variants A) and B) for ESP variants C) and D). [file 1471-2164-16-S8-S2-S12.pdf]

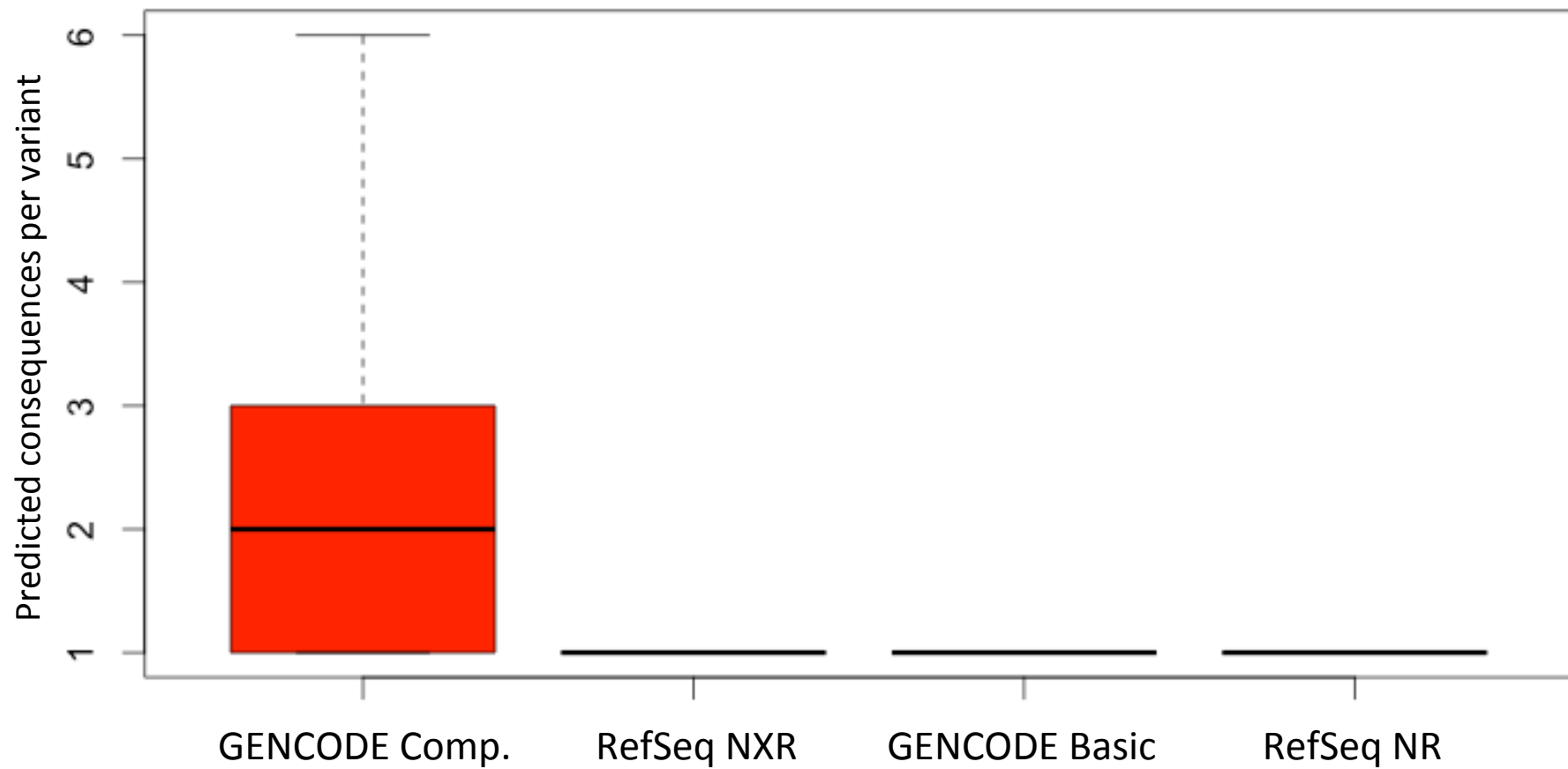

Supplement: Additional file 14 — Figure S9 - Number of predicted consequences per variant. Box plot of the number of predicted consequences for each variant by geneset. [file 1471-2164-16-S8-S2-S14.pdf]
