# Supplementary material for: Comparison of GENCODE and RefSeq gene annotation and the impact of reference geneset on variant effect prediction
Source: BMC Genomics. 2015 Jun 18;16(Suppl 8):S2. doi: 10.1186/1471-2164-16-S8-S2 (PMC4502323; doi:10.1186/1471-2164-16-S8-S2)
Supplement: Additional file 10 — Table S4 - Derivation of broad variant classes. Derivation of grouping of broad variant classes from functional annotation terms from VEP. [file 1471-2164-16-S8-S2-S10.pdf]

Variant Class	Ensembl VEP annotation included
LoF	stop_gained<br>splice_donor_variant<br>splice_acceptor_variant<br>frameshift_variant
CDS	initiator_codon_variant<br>stop_lost<br>inframe_deletion<br>stop_retained_variant<br>inframe_insertion<br>coding_sequence_variant<br>incomplete_terminal_codon_variant<br>missense_variant<br>synonymous_variant
splice	splice_region_variant
other	5_prime_UTR_variant<br>3_prime_UTR_variant<br>non_coding_transcript_exon_variant
