# Supplementary material for: Comparison of GENCODE and RefSeq gene annotation and the impact of reference geneset on variant effect prediction
Source: BMC Genomics. 2015 Jun 18;16(Suppl 8):S2. doi: 10.1186/1471-2164-16-S8-S2 (PMC4502323; doi:10.1186/1471-2164-16-S8-S2)
Supplement: Additional file 13 — Dominant expression analysis. Results and methods for the analysis of dominantly expressed GENCODE transcripts. [file 1471-2164-16-S8-S2-S13.pdf]

Using dominant transcript expression to further refine the GENCODE geneset

The introduction of the GENCODE Basic geneset as a simple filtering option reduces the complexity of the annotation presented to the user and thus simplifies the interpretation of variant consequences. In order to try and simplify the transcript set further and make the restricted set more biologically relevant, we considered using RNAseq to identify and prioritise transcripts according to their expression level in a tissue, cell-line or experimental condition of interest. However, the ability of Illumina RNAseq data to achieve the accurate transcript-level reconstruction and quantification necessary for such filtering has been called into question[1, 2], typically on the basis that the relatively short read length makes it difficult to reliably resolve individual transcripts at loci with multiple AS events. Having excluded this option, we investigated the possibility of using RNAseq data to tackle the more tractable problem of identifying the most highly expressed transcript of a locus in any experiment[3]. We used FluxCapacitor[4] and Cufflinks2[5] to independently identify dominant transcripts in 154 ENCODE 2 RNAseq datasets. This allowed a comparison of their results and an assessment of whether they could provide a reliable means of specific, biologically informed filtering of the GENCODE transcript set. Across the 154 datasets Flux Capacitor and Cufflinks2 both identified a dominant transcript (although not necessarily the same transcript) for between 50% and 60% of all genes in any one experiment. However, where Flux Capacitor and Cufflinks2 do both identify a dominant transcript, they agree in up to 90% of cases (in the case of 5x dominance i.e. where the dominant transcript is expressed at a level >5x greater than the next highest) (Additional file 15: Figure S10). Where both methods agree

on a dominant transcript, it is likely (75-90%) to be in the GENCODE Basic set (Additional file 16: Figure S11). This suggests that the GENCODE Basic set contains a large proportion of the most highly expressed transcripts, even though it was not derived using transcript quantification. As it is difficult to obtain reference transcript quantification to validate these predictions, we used an orthogonal dataset. The APPRIS pipeline[6] uses information about protein structure and function, along with cross-species conservation to identify the CDS representing the ‘principal isoform’, presumed to be the main functional product of the locus. Predictions of the dominant transcript by both methods (and their intersection) share a high degree of overlap (mean ~71%) with the ‘principal isoform’ predicted by APPRIS) (Additional file 17: Figure S12). This suggests that, given the restrictions of current RNAseq data and transcript quantification methods, the predictions form a reasonable, if not perfect, basis to support an additional option for selecting a representative transcript from any given locus, particularly when used in combination with each other and the APPRIS principal isoform data.

Dominant transcript analysis

154 paired-end libraries from the Encode Cell Lines experiment (http://www.ebi.ac.uk/gxa/experiments/E-GEOD-26284) were mapped against human genome reference from Ensembl 73 using Tophat2[7], and then quantified transcript expression using Cufflinks2 and Flux Capacitor v.1.6.1, obtaining an FPKM value for each gene-library pair. For each library and gene a dominant transcript was identified if a. it was the only transcript for that gene or b. if its FPKM expression was at least N-fold

higher than that of the second highest expressed transcript (where N=0 (i.e. no dominance but expression difference) N = 2 or N = 5). A minimum expression level cut-offs to separate 'real' expression from 'background' was also applied: FPKM=0, 2 and 5 (where 0 means that all data was included). Predicted dominant transcripts were compared with predicted principal isoforms from the APPRIS pipeline (release corresponding to Ensembl 73).

1. Tilgner H, Raha D, Habegger L, Mohiuddin M, Gerstein M, Snyder M: **Accurate identification and analysis of human mRNA isoforms using deep long read sequencing**. *G3* 2013, **3**(3):387-397.
2. Steijger T, Abril JF, Engstrom PG, Kokocinski F, Hubbard TJ, Guigo R, Harrow J, Bertone P, Consortium R: **Assessment of transcript reconstruction methods for RNA-seq**. *Nature methods* 2013, **10**(12):1177-1184.
3. Gonzalez-Porta M, Frankish A, Rung J, Harrow J, Brazma A: **Transcriptome analysis of human tissues and cell lines reveals one dominant transcript per gene**. *Genome biology* 2013, **14**(7):R70.
4. **Flux Capacitor** [http://sammeth.net/confluence/display/FLUX/Home]
5. Trapnell C, Williams BA, Pertea G, Mortazavi A, Kwan G, van Baren MJ, Salzberg SL, Wold BJ, Pachter L: **Transcript assembly and quantification by RNA-Seq reveals unannotated transcripts and isoform switching during cell differentiation**. *Nature biotechnology* 2010, **28**(5):511-515.
6. Rodriguez JM, Maietta P, Ezkurdia I, Pietrelli A, Wesselink JJ, Lopez G, Valencia A, Tress ML: **APPRIS: annotation of principal and alternative splice isoforms**. *Nucleic acids research* 2013, **41**(Database issue):D110-117.
7. Kim D, Pertea G, Trapnell C, Pimentel H, Kelley R, Salzberg SL: **TopHat2: accurate alignment of transcriptomes in the presence of insertions, deletions and gene fusions**. *Genome biology* 2013, **14**(4):R36.
