# Supplementary material for: Comparison of GENCODE and RefSeq gene annotation and the impact of reference geneset on variant effect prediction
Source: BMC Genomics. 2015 Jun 18;16(Suppl 8):S2. doi: 10.1186/1471-2164-16-S8-S2 (PMC4502323; doi:10.1186/1471-2164-16-S8-S2)
Supplement: Additional file 15 — Figure S10 - Comparison of dominant transcript calls between FluxCapacitor and Cufflinks2. Percentage of agreement between dominant transcripts assigned by FluxCapacitor and Cufflinks2 at all protein genes across 154 ENCODE 2 cell lines. [file 1471-2164-16-S8-S2-S15.pdf]

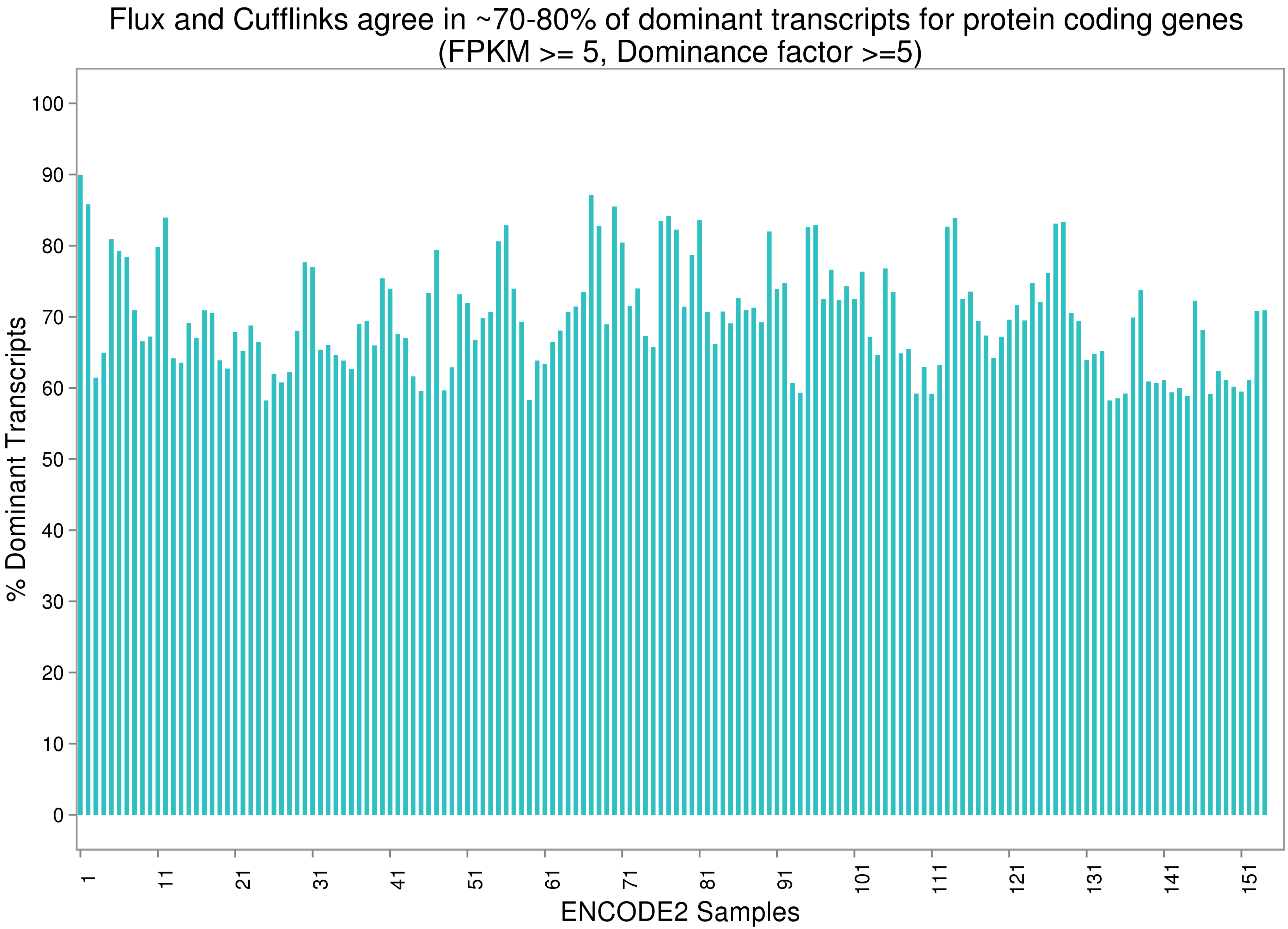
Flux and Cufflinks agree in ~70-80% of dominant transcripts for protein coding genes
(FPKM >= 5, Dominance factor >=5)
% Dominant Transcripts
0
10
20
30
40
50
60
70
80
90
100
1
11
21
31
41
51
61
71
81
91
101
111
121
131
141
151
ENCODE2 Samples
