# Supplementary material for: Comparison of GENCODE and RefSeq gene annotation and the impact of reference geneset on variant effect prediction
Source: BMC Genomics. 2015 Jun 18;16(Suppl 8):S2. doi: 10.1186/1471-2164-16-S8-S2 (PMC4502323; doi:10.1186/1471-2164-16-S8-S2)
Supplement: Additional file 16 — igure S11 - Comparison of dominant transcript calls from FluxCapacitor and Cufflinks2 with APPRIS pipeline. Percentage of agreement between dominant transcripts assigned by FluxCapacitor and Cufflinks2 and APPRIS principal isoforms at all protein genes across 154 ENCODE 2 cell lines. Dominant transcripts reported by Cufflinks2(Grey bars) and FluxCapacitor(Blue) are shown. [file 1471-2164-16-S8-S2-S16.pdf]

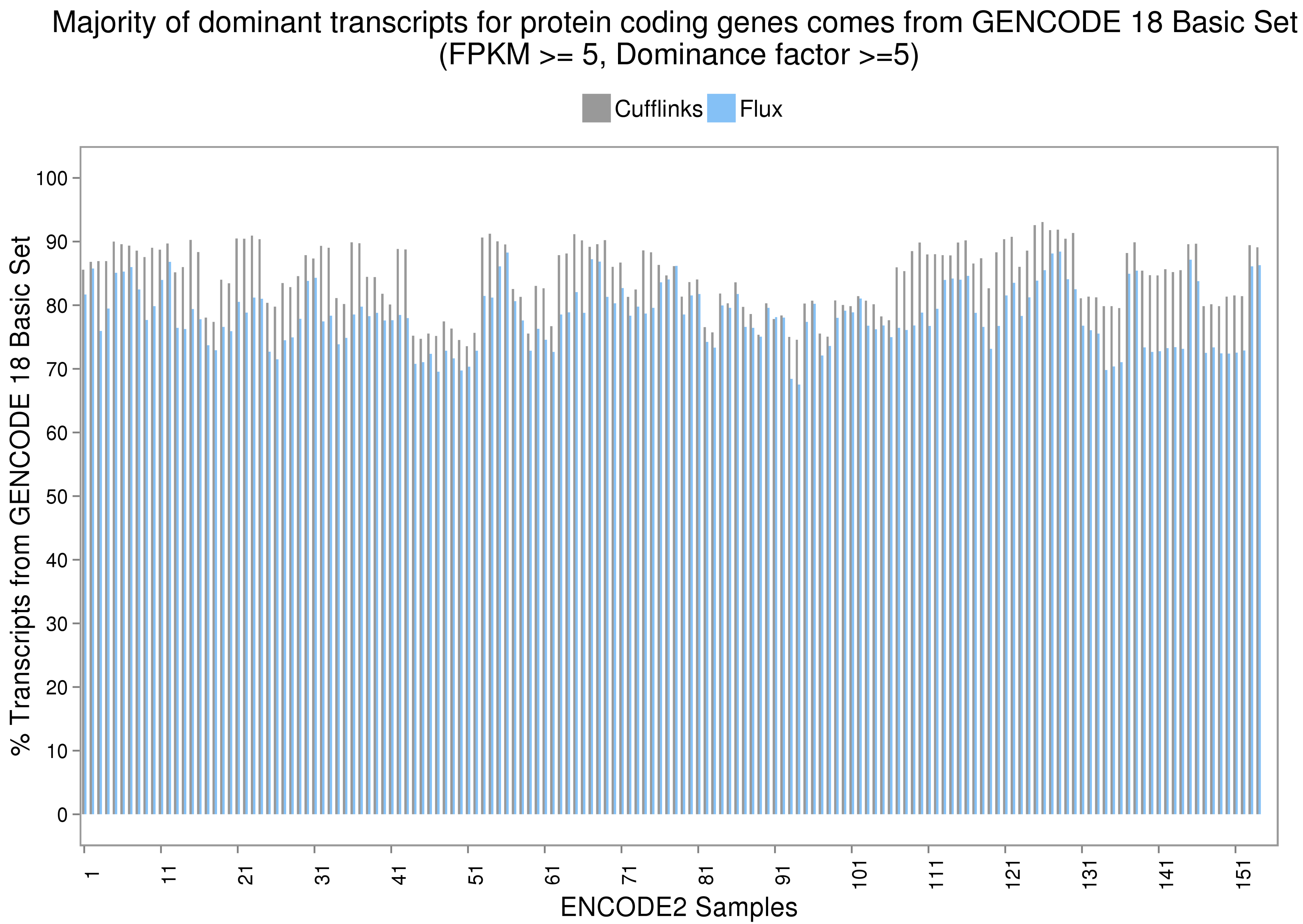

Majority of dominant transcripts for protein coding genes comes from GENCODE 18 Basic Set
(FPKM >= 5, Dominance factor >=5)
Cufflinks
Flux
% Transcripts from GENCODE 18 Basic Set
0
10
20
30
40
50
60
70
80
90
100
1
11
21
31
41
51
61
71
81
91
101
111
121
131
141
151
ENCODE2 Samples
