# Supplementary material for: Comparison of GENCODE and RefSeq gene annotation and the impact of reference geneset on variant effect prediction
Source: BMC Genomics. 2015 Jun 18;16(Suppl 8):S2. doi: 10.1186/1471-2164-16-S8-S2 (PMC4502323; doi:10.1186/1471-2164-16-S8-S2)
Supplement: Additional file 17 — Figure S12 - Comparison of dominant transcript calls from FluxCapacitor and Cufflinks2 with GENCODE Basic geneset. Percentage of agreement between dominant transcripts assigned by FluxCapacitor and Cufflinks2 and GENCODE Basic transcripts at all protein genes across 154 ENCODE 2 cell lines. Dominant transcripts reported by Cufflinks2(Grey bars) and FluxCapacitor(Blue) are shown. [file 1471-2164-16-S8-S2-S17.pdf]

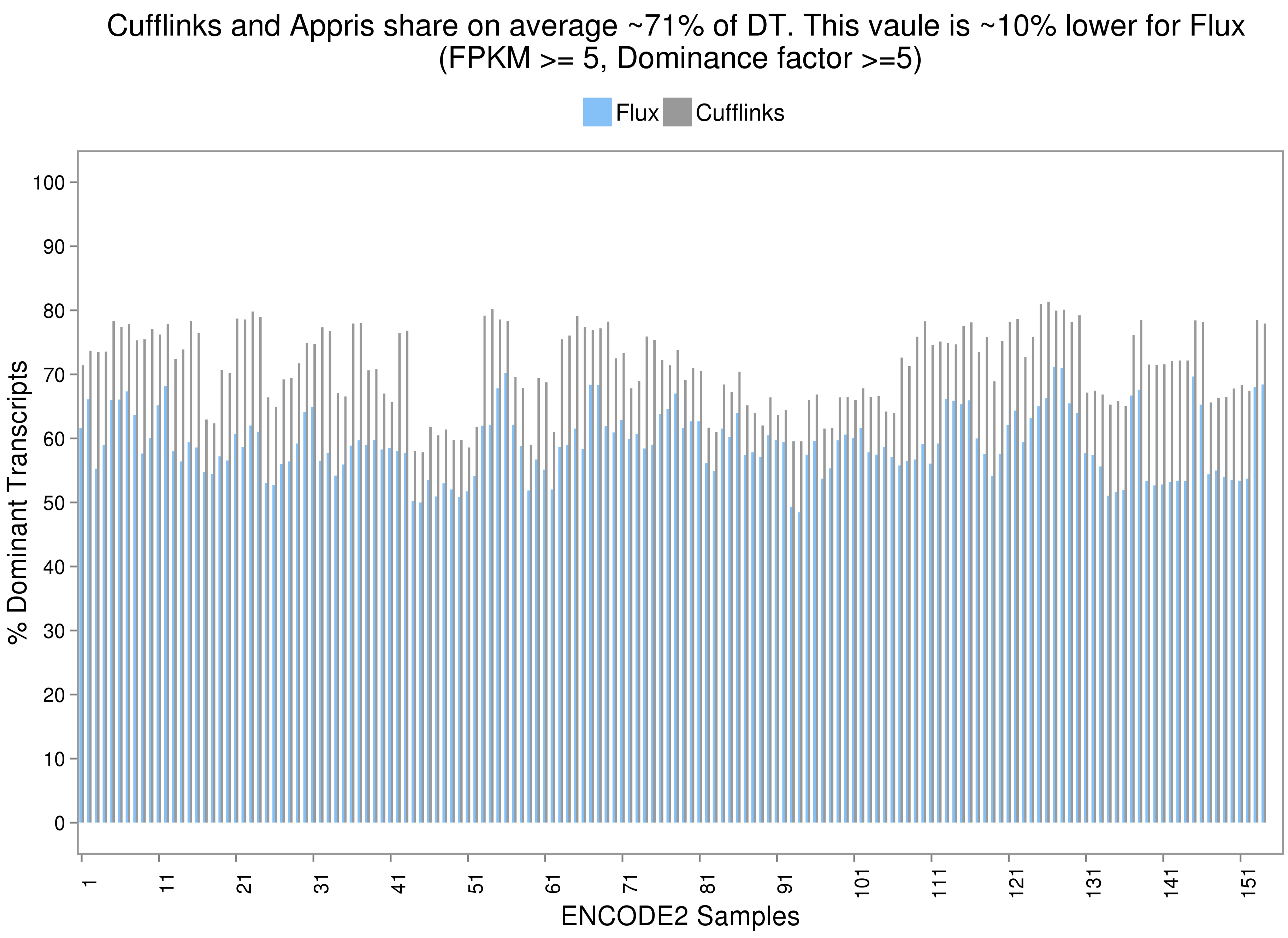

Cufflinks and Appris share on average ~71% of DT. This vaule is ~10% lower for Flux
(FPKM >= 5, Dominance factor >=5)
Flux
Cufflinks
% Dominant Transcripts
0
10
20
30
40
50
60
70
80
90
100
1
11
21
31
41
51
61
71
81
91
101
111
121
131
141
151
ENCODE2 Samples
