# Supplementary material for: Comparison of GENCODE and RefSeq gene annotation and the impact of reference geneset on variant effect prediction
Source: BMC Genomics. 2015 Jun 18;16(Suppl 8):S2. doi: 10.1186/1471-2164-16-S8-S2 (PMC4502323; doi:10.1186/1471-2164-16-S8-S2)
Supplement: Additional file 1 — Figure S1 - Comparison of GENCODE and RefSeq Annotation in the Ensembl genome browser. Screenshot of the Ensembl genome browser displying the BRCA1 locus. GENCODE gene annotation is shown at the top of the panel. Manually annoated protein-coding transcripts are shown in red, manually annotated NMD, processed_transcript and retained_intron transcripts are shown in royal blue and merged manual and computational transcripts are shown in gold. RefSeq transcripts are shown in dark blue. Computationally predicted XM transcripts are highlighted by blue arrowheads, manually annotated, protein-coding NM transcripts in green and manually annotated non-coding NR transcripts in red. [file 1471-2164-16-S8-S2-S1.pdf]

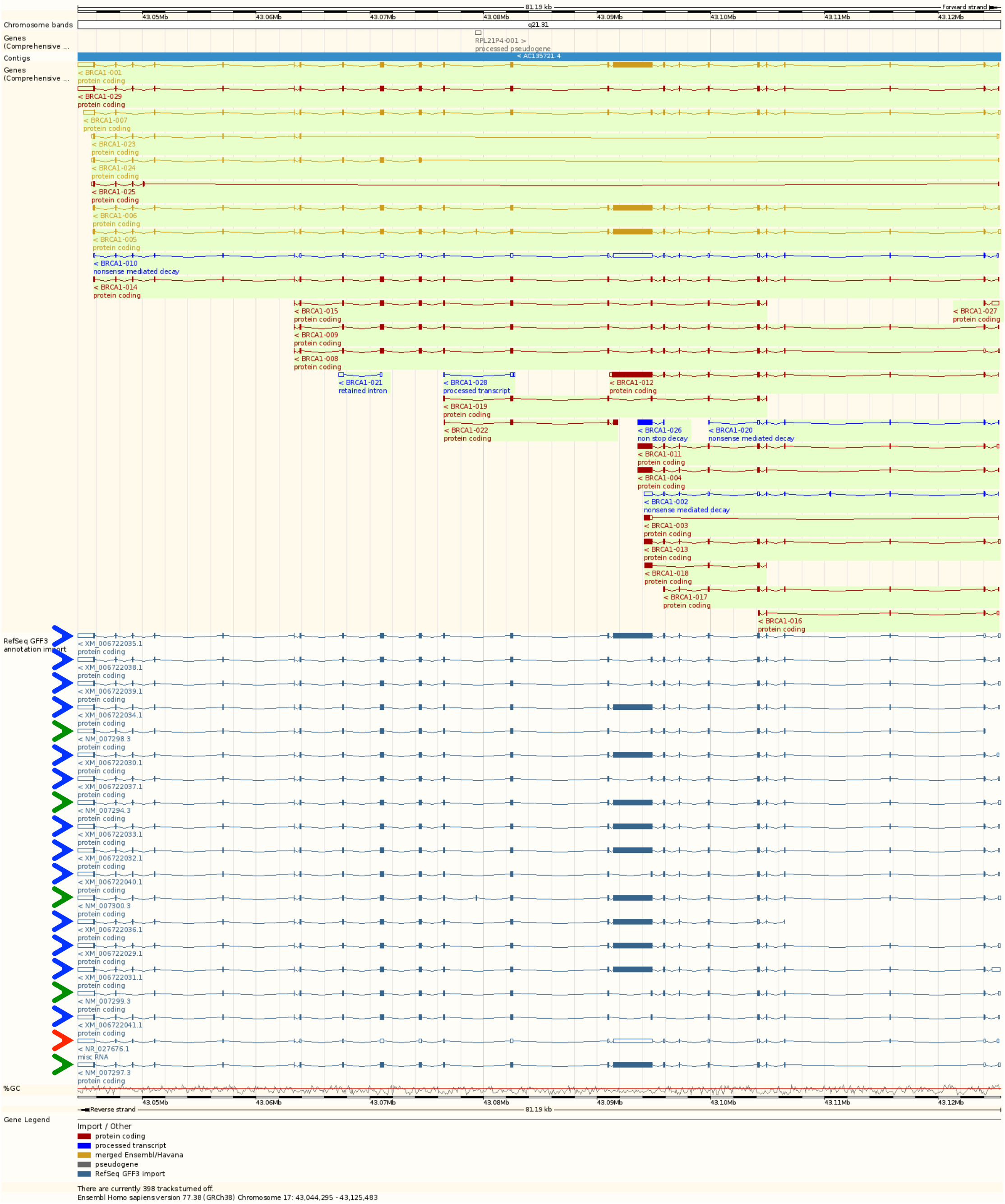

81.19 kb
Forward strand
43.05Mb
43.06Mb
43.07Mb
43.08Mb
43.09Mb
43.10Mb
43.11Mb
43.12Mb
Chromosome bands
q21.31
Genes (Comprehensive ...
RPL21P4-001 >
processed pseudogene
Contigs
< AC135721.4
Genes (Comprehensive ...
< BRCA1-001 protein coding
< BRCA1-029 protein coding
< BRCA1-007 protein coding
< BRCA1-023 protein coding
< BRCA1-024 protein coding
< BRCA1-025 protein coding
< BRCA1-006 protein coding
< BRCA1-005 protein coding
< BRCA1-010 nonsense mediated decay
< BRCA1-014 protein coding
< BRCA1-015 protein coding
< BRCA1-027 protein coding
< BRCA1-009 protein coding
< BRCA1-008 protein coding
< BRCA1-021 retained intron
< BRCA1-028 processed transcript
< BRCA1-012 protein coding
< BRCA1-019 protein coding
< BRCA1-022 protein coding
< BRCA1-026 non stop decay
< BRCA1-020 nonsense mediated decay
< BRCA1-011 protein coding
< BRCA1-004 protein coding
< BRCA1-002 nonsense mediated decay
< BRCA1-003 protein coding
< BRCA1-013 protein coding
< BRCA1-018 protein coding
< BRCA1-017 protein coding
< BRCA1-016 protein coding
RefSeq GFF3 annotation import
< XM_006722035.1 protein coding
< XM_006722038.1 protein coding
< XM_006722039.1 protein coding
< XM_006722034.1 protein coding
< NM_007298.3 protein coding
< XM_006722030.1 protein coding
< XM_006722037.1 protein coding
< NM_007294.3 protein coding
< XM_006722033.1 protein coding
< XM_006722032.1 protein coding
< XM_006722040.1 protein coding
< NM_007300.3 protein coding
< XM_006722036.1 protein coding
< XM_006722029.1 protein coding
< XM_006722031.1 protein coding
< NM_007299.3 protein coding
< XM_006722041.1 protein coding
< NR_027676.1 misc RNA
< NM_007297.3 protein coding
%GC
Reverse strand
Gene Legend
Import / Other
protein coding
processed transcript
merged Ensembl/Havana
pseudogene
RefSeq GFF3 import
There are currently 398 tracks turned off.
Ensembl Homo sapiens version 77.38 (GRCh38) Chromosome 17: 43,044,295 - 43,125,483
