# Supplementary material for: Comparison of GENCODE and RefSeq gene annotation and the impact of reference geneset on variant effect prediction
Source: BMC Genomics. 2015 Jun 18;16(Suppl 8):S2. doi: 10.1186/1471-2164-16-S8-S2 (PMC4502323; doi:10.1186/1471-2164-16-S8-S2)
Supplement: Additional file 2 — Figure S2 - Comparison of GENCODE and RefSeq Annotation in the UCSC genome browser. Screenshot of the UCSC genome browser displying the SLC25A17 locus. RefSeq gene annotation is shown in dark blue in the top panel. GENCODE gene annotation is shown in the middle panel in blue (protein-coding and NMD transcripts) and green (processed_transcripts). CCDS transcripts are shown in the lowest panel in green. Novel GENCODE splicing features are highlighted in red (novel cassette exons), blue (novel alternative splice site, or shifted splice site) and green (novel putative TSS and 5' UTR). [file 1471-2164-16-S8-S2-S2.pdf]

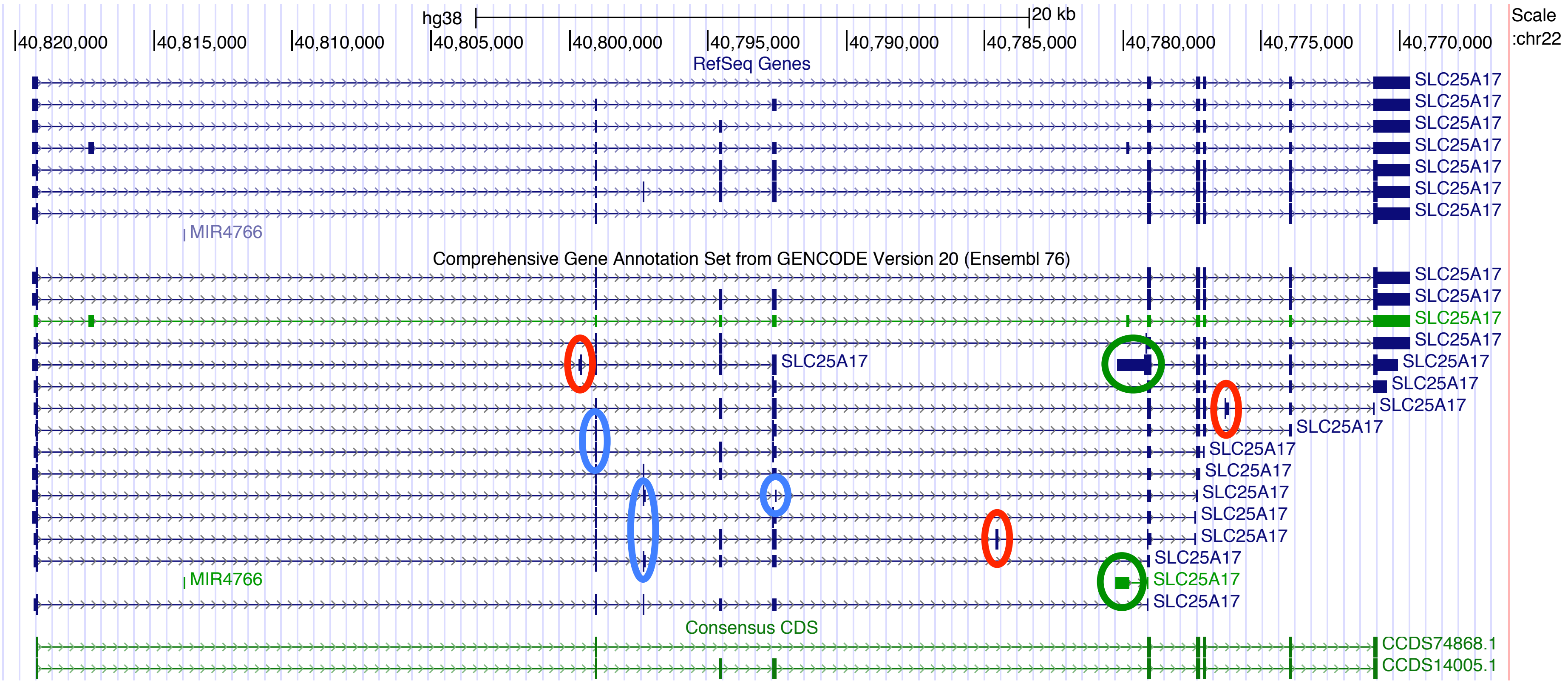

Novel cassette exon

Novel alternative splice site

Novel putative TSS, 5’ UTR
